# Oligophenyls with Multiple Disulfide Bridges as Higher Homologues of Dibenzo[*c*,*e*][1,2]dithiin: Synthesis and Application in Lithium‐Ion Batteries

**DOI:** 10.1002/chem.202000728

**Published:** 2020-06-05

**Authors:** Christoph Sonnenschein, Christopher P. Ender, Faxing Wang, Dieter Schollmeyer, Xinliang Feng, Akimitsu Narita, Klaus Müllen

**Affiliations:** ^1^ Planck Institute for Polymer Research Ackermannweg 10 55128 Mainz Germany; ^2^ Center for Advancing Electronics Dresden (cfaed) and Faculty of Chemistry and Food Chemistry Chair for Molecular Functional Materials Technische Universität Dresden 01062 Dresden Germany; ^3^ Institut für Organische Chemie Johannes Gutenberg-Universität Mainz Duesbergweg 10–14 55099 Mainz Germany

**Keywords:** dibenzo[1,2]dithiin, lithium-ion batteries, oligophenyls, polycycles, sulfur heterocycles

## Abstract

Higher homologues of dibenzo[*c*,*e*][1,2]dithiin were synthesized from oligophenyls bearing multiple methylthio groups. Single‐crystal X‐ray analyses revealed their nonplanar structures and helical enantiomers of higher *meta*‐congener **6**. Such dibenzo[1,2]dithiin homologues are demonstrated to be applicable to lithium‐ion batteries as cathode, displaying a high capacity of 118 mAh g^−1^ at a current density of 50 mA g^−1^.

## Graphical abstract

**Oligophenyls** with multiple disulfide bridges were synthesized as higher homologues of dibenzo[*c*,*e*][1,2]dithiin, through demethylation and oxidative disulfide bond formation of methylthiolated oligophenyls. Such homologues of dibenzo[1,2]dithiin homologues can serve as cathode material in lithium‐ion, displaying a high capacity of 118 mAh g^−1^ at a current density of 50 mA g^−1^.

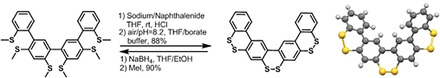

Substituted and *ortho*‐bridged biphenyls play an important role, for example, as models for stereochemical studies,^1^ natural products^2^ and chiral ligands of metal catalysts.^3^ When looked upon from a different angle, biphenyls and their higher oligophenyl homologues are conjugated π‐systems with useful electronic properties.^4^ A special, though rarely studied, case are biphenyls, oligophenyls, and polycyclic aromatic hydrocarbons, carrying disulfide bridges, such as 4,5,9,10‐tetrathiapyrene (**1**) and dinaphtho[1,2]dithiin (**2**) having dibenzo[*c*,*e*][1,2]dithiin (**3**) as substructure (see Figure 1).^5^

**Figure 1:**
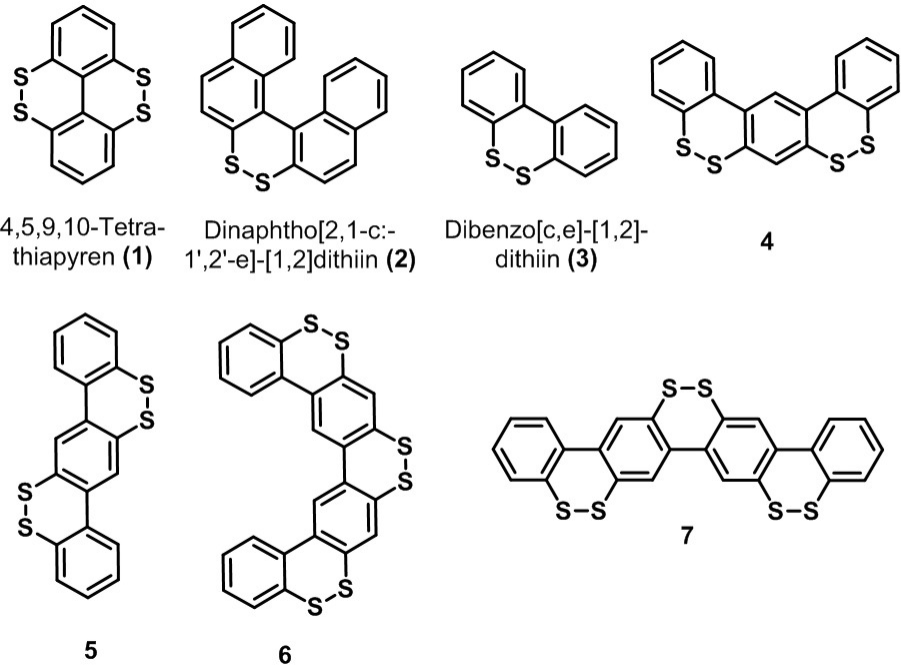
Examples of known dibenzo[1,2]dithiin derivatives:^5^ tetrathiapyrene **1**, dinaphtho‐ and dibenzo[1,2]dithiin **2** and **3**, respectively, as well as dibenzo[1,2]dithiin homologues **4**–**7** synthesized in this work.

One expects a pronounced influence of the sulfur (S) substituents upon electron transfer processes such as occurring upon the charging/discharging of battery elements.^6^ In conventional sulfur cathodes of lithium (Li)‐ion batteries, lithium polysulfide intermediates generated during discharge are highly soluble in the electrolyte, and can freely diffuse to the anode, irreversibly reacting with the Li metal.^6^ To prevent the diffusion of lithium polysulfide intermediates and the resulting deterioration of the batteries, one promising approach is to use organic cathode materials with covalently bonded disulfides, which can undergo intramolecular redox reactions with the Li ions (C‐S‐S‐C+2 Li^+^+2 e^−^⇄2C‐S‐Li).^6^, ^7^ For example, poly[bis(2‐aminophenyloxy)disulfide]7a and poly(tetrathionaphthalene)7b are demonstrated to be applicable as electrode materials in lithium‐ion batteries. Nevertheless, such sulfur‐rich organic cathode materials remain underexplored.

On the other hand, disulfide bridges are known to play an important role in controlling conformations, with the classical example coming from the peptide chemistry.^8^ There, oxidative formation of disulfide bonds from cysteine thiol groups is essential in establishing the 3D‐structure of the biomolecules.^8^ In case of oligophenyl systems, we considered that electron‐transfer‐induced opening or closing of intramolecular S−S bonds^9^ could also lead to conformation control. Nevertheless, oligophenyls with multiple fused [1,2]dithiin rings, namely higher homologues of dibenzo[*c*,*e*][1,2]dithiin (**3**), have not been reported.^5^

Herein, we describe syntheses of a series of dibenzo[1,2]dithiin homologues **3**–**7** using oligophenyl precursors **8**–**12** bearing multiple methylthio groups. The key step comprises reductive cleavage of alkyl‐sulfur bonds followed by oxidative S−S bond formation. To the best of our knowledge, similar protocols have never been applied toward syntheses of such dibenzo[1,2]dithiins. The geometric structures of **4**–**6** are elucidated by single‐crystal X‐ray diffraction, in particular, demonstrating helical enantiomers of **6**. Furthermore, we have investigated the applicability of **4** and **6** in lithium‐ion batteries.^10^ Dibenzo[1,2]dithiin **6** exhibits a specific capacity of 118 mAh g^−1^ and an energy density of 236 Wh kg^−1^, which is highly competitive with previously reported sulfur‐rich organic electrode materials for lithium‐ion batteries.^5^, ^6^, ^7^

2,2′‐Bis(methylthio)‐1,1′‐biphenyl (**8**) was initially synthesized by a Suzuki coupling of bromo‐2‐(methylthio)‐benzene (**13**) and 2‐(methylthio)‐benzene‐boronic acid (**14**) in 92 % yield (Figure 2). Syntheses of *o*‐methylthiolated‐*m*‐oligophenyls **9** and **11** were also carried out by using the Suzuki coupling (Figure 2). *m*‐Dibromo‐bis(methylthio)benzene **15** 11 was reacted with 2‐(methylthio)‐phenylpinacolborate (**16**) under Suzuki conditions to afford tetrakis(methylthio)‐*m*‐terphenyl **9** in 84 % yield. For the synthesis of hexakis(methylthio)‐*m*‐quaterphenyl **11**, compound **15** was treated with *n‐butyl*lithium at −100 °C, and the resulting lithiated intermediate was quenched with 2‐isopropoxy‐4,4,5,5‐tetramethyl‐1,3,2‐dioxaborolane (PinBO*i*Pr) or iodine to obtain boronic ester **17** and iodide **18**, respectively. Suzuki coupling of **17** and **18** yielded dibromo‐biphenyl **19** in 65 % yield, which was further subjected to a twofold Suzuki reaction with **16** to afford **11** in 78 % yield.

**Figure 2:**
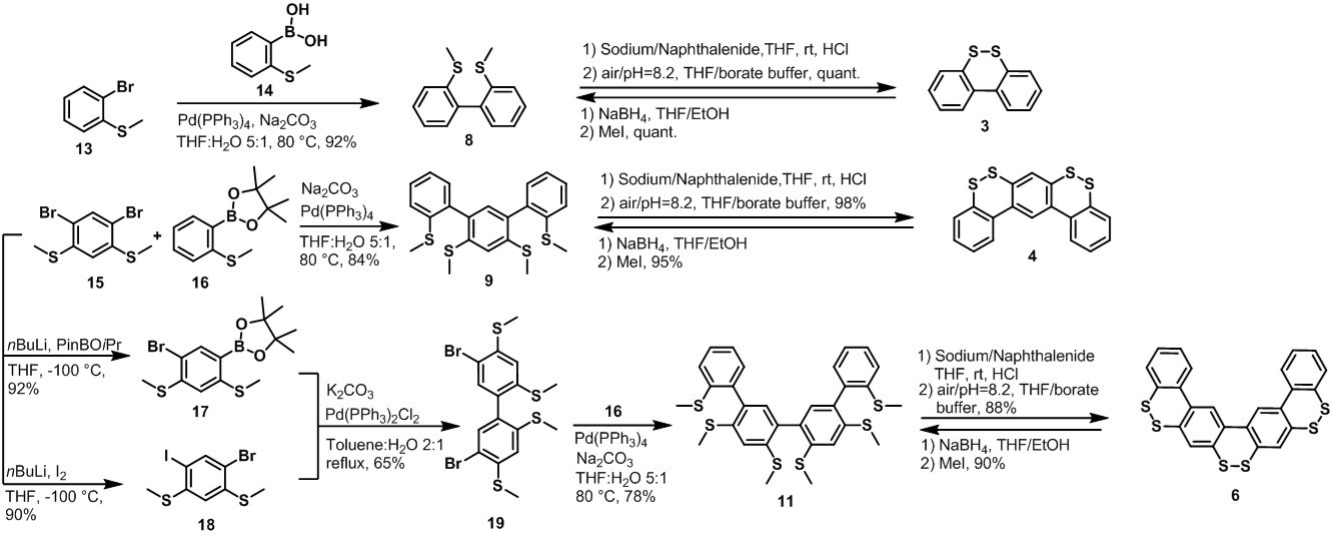
Synthesis of dibenzo[1,2]dithiin homologues **3**, **4** and **6**.

The syntheses of *o*‐methylthiolated‐*p*‐oligophenyls **10** and **12** were carried out as displayed in Figure 3. Suzuki coupling of *p*‐dibromo‐bis(methylthio)benzene **20** 12 did not proceed with boronic ester **16**, but the use of boronic acid **14** successfully provided **10** in 90 % yield. Iodide **21** and boronic acid **22** were synthesized from **20** in similar manners as the preparations of **18** and **17**, respectively, employing B(OMe)_3_ instead of PinBO*i*Pr. Suzuki reaction of **21** and **22** then afforded dibromo‐biphenyl **23** in 82 % yield. Finally, twofold coupling of **23** with **14** provided hexakis(methylthio)‐*p*‐quaterphenyl **12** in 85 % yield.

**Figure 3:**
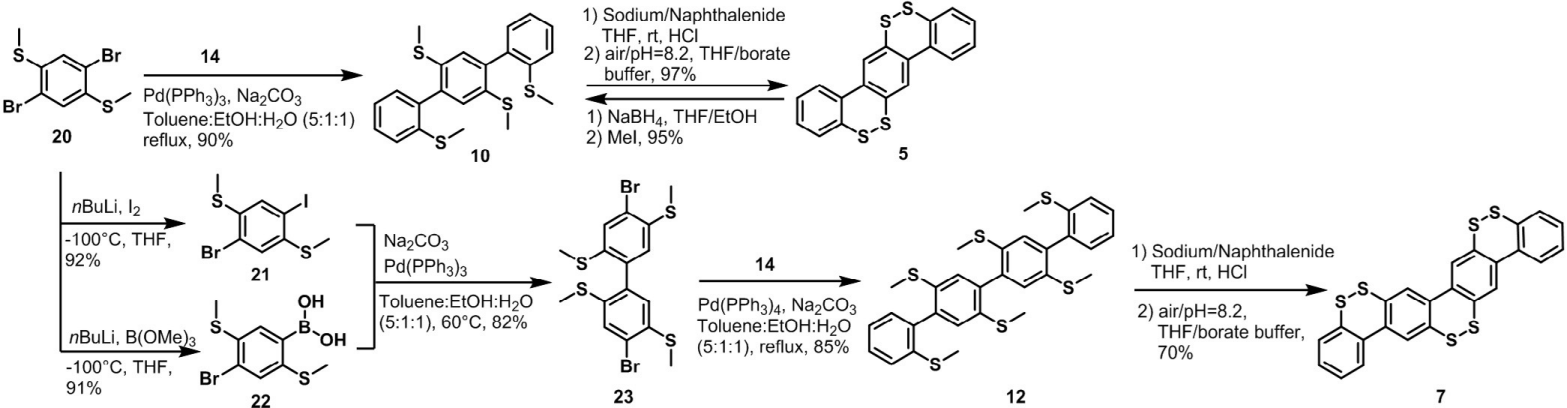
Synthesis of dibenzo[1,2]dithiin homologues **5** and **7**.

Finally, methylthiolated oligophenyls **8**–**12** were subjected to reductive demethylation and protonation via hydrogen chloride to afford the corresponding intermediate thiol oligophenyl compounds. After dissolving these thiols in degassed mixtures of THF/water (10:1) and adjusting the pH to 8.2 with sodium borate buffer, the solutions were exposed to air to allow for oxidative S−S bond formation. The resulting dibenzo[1,2]dithiin homologues **3**–**7** could be purified by column chromatography over silica gel and characterized by ^1^H and ^13^C NMR spectroscopy as well as high‐resolution mass spectrometry (see Figure 4 and Supporting Information). Dibenzo[1,2]dithiins **3**–**5** could be obtained in 97 % to quantitative yields. Nevertheless, the yields of forming higher homologous **6** and **7** from **11** and **12** were compromised, namely 88 % and 70 %, respectively, due to partial loss of sulfur under the oxidative condition, resulting in the formation of dibenzothiophene moieties (Figure S4, Supporting Information). With the help of nuclear Overhauser enhancement spectroscopy (NOESY), the aromatic proton signals could be clearly assigned, which further provided the structure proof (spectra of **6** as example in Figure 4 a). Moreover, single crystals suitable for X‐ray diffraction analysis were obtained for **4**–**6**, **9** and **10**.

**Figure 4:**
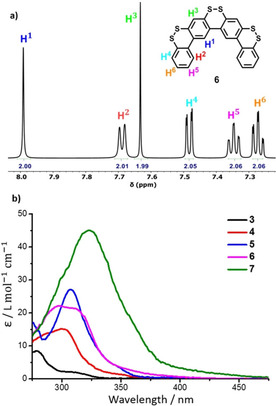
a) ^1^H‐NMR of *m*‐quaterphenyl‐tris(dithiin) **6** and b) UV/Vis spectra of **3**–**7** in THF (10^−5^ m).

Comparison of the single‐crystal structures of **9** and **4** revealed that the *meso*‐isomer of **9** (Figure 5 a) was maintained upon ring closure, leading to the *meso*‐configuration of **4** (Figure 5 b). One can thus assume that this is the thermodynamically favored configuration in the solid state. The dihedral angle of the biphenyl unit was reduced from 85° (**9**) to 35° (**4**) upon the formation of the disulfide bonds. Similarly, the twist of the biphenyl units in **10** (76°, Figure 5 c) was decreased to 31° and 26° in **5** (Figure 5 d) while maintaining the *meso*‐configuration as well. In contrast, a racemic mixture was observed in the single‐crystal structure of the higher homologue **6**, displaying the co‐crystallization of two enantiomers (Figure 5 e).

**Figure 5:**
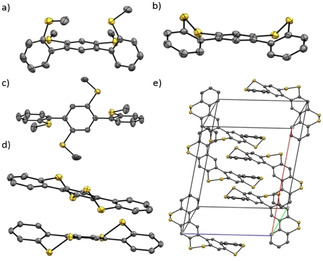
Crystal structure of **9**; b) Crystal structure of **4**; c) Crystal structure of **10**; d) Crystal structure of **5**; e) Racemic crystal structure of **6** (ORTEP‐Plots showing 50 % thermal ellipsoid probabilities).

UV/Vis absorption spectra of dibenzo[1,2]dithiin homologues **3**–**7** revealed redshifts upon structural extension, indicating lowering of optical energy gaps (Figure 4 b). In case of *meta*‐congeners **4** and **6**, the redshift was small, with the absorption edge moving from 325 nm (**4**) to 332 nm (**6**), corresponding to a decrease of the optical gap from 3.81 eV to 3.73 eV. In contrast, *para*‐congeners **5** and **7** demonstrated more significant redshifts of the absorption peaks, exhibiting the change of the absorption onset from **5**: 336 nm (3.69 eV) to **7**: 370 nm (3.35 eV). As expected, these results indicated more effective extension of the π‐conjugation in the *para*‐congeners than in the *meta*‐congeners.

Additionally, the possibility to reopen the formed dibenzo[1,2]dithiins **3**–**7** was examined by reduction with NaBH_4_ in ethanol/THF (1:1) followed by protection of the resulting thiols with an excess of methyl iodide. Although the low solubility of **7** did not allow the ring‐opening back to **12**, thioethers **8**–**11** were obtained in 90 % to quantitative yields from **3**–**6**, respectively. These results indicated the chance of reversibly closing and reopening the multiple disulfide bonds, and also motivated us to investigate the applicability of such dibenzo[1,2]dithiin homologues as electrode materials for Li‐ion batteries. We selected dibenzo[1,2]dithiins **4** and **6** as representative homologues and investigated their electrochemical performance for Li‐ion battery applications by cyclic voltammetry (CV) in 1 m LiCF_3_SO_3_ (LiTFSI) electrolyte and by galvanostatic charge/discharge profiles in the voltage range of 1.5–3.0 V. CV curves of both **4** and **6** (Figure 6 a and Figure S5 a) displayed one pair of redox peaks at 1.95 V in the cathodic process and 2.55 V in the anodic process, corresponding to formation of C‐S‐Li complexes through the opening of the S−S bonds.^6^ The large voltage difference in the cathodic and anodic process indicates a large electrode polarization, which is caused by the low electronic conductivity of the dibenzo[1,2]dithiin‐based electrode.^7^ From the galvanostatic charge/discharge profiles, dibenzo[1,2]dithiin **6** exhibited a specific capacity of 118 mAh g^−1^ at a current density of 50 mA g^−1^ (Figure 6 b), which was higher than that of **4** (85 mAh g^−1^) (Figure S5 b). The higher specific capacity of dibenzo[1,2]dithiin **6** compared to **4** can be ascribed to the higher sulfur content per molecule of **6**. When current density was increased to 75, 100 and 150 mA g^−1^, dibenzo[1,2]dithiin **6** still delivered specific capacities of 110, 104 and 84 mA g^−1^, respectively. The average discharge voltage plateau was approximately 2 V at 50 mA g^−1^, producing an energy density of about 236 Wh kg^−1^ based on the mass of **6**. The specific capacities and energy densities of dibenzo[1,2]dithiin **6** are highly competitive with those of other reported sulfur‐rich organic electrode materials, such as poly[bis(2‐aminophenyloxy)disulfide] (50–200 mAh g^−1^),7a poly(5,8‐dihydro‐1 H,4*H*‐2,3,6,7‐tetrathia‐anthracene) (29–170 mAh g^−1^)7c and poly(tetrathionaphthalene) (101–120 mAh g^−1^).7b Moreover, after 30 cycles, the specific capacity of dibenzo[1,2]dithiin **4** was stabilized at around 74 mAh g^−1^, corresponding to 86 % capacity retention and a small capacity fading of only 0.46 % per cycle (Figure S5 c). Similarly, dibenzo[1,2]dithiin **6** showed a capacity retention of 84 % after 30 cycles (Figure S5 d). In addition, a charged Li‐ion battery prepared with dibenzo[1,2]dithiin **6** as the cathode could readily power 33 commercial red light‐emitting diodes (LED, 1.7–2.3 V) in parallel connection (Figure S6) for twenty minutes (see Supporting Information for details), demonstrating the high potential of such dibenzo[1,2]dithiin homologues in Li‐ion batteries.

**Figure 6:**
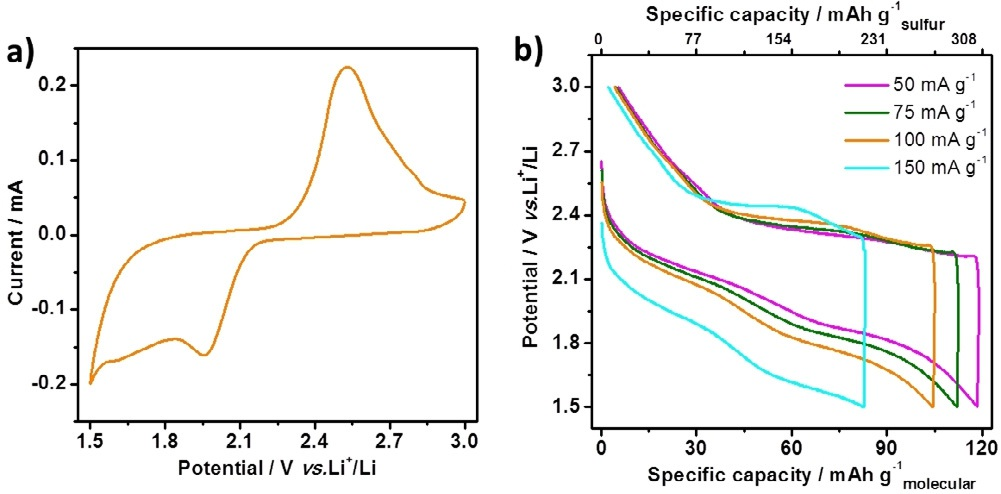
a) The CV curves of **6** in the voltage range of 1.5–3.0 V; b) The galvanostatic charge/discharge curves of **6** at various current densities.

In summary, we have achieved the multistep syntheses of methylthiolated oligophenyls **8**–**12** and the subsequent formation of disulfide bonds to obtain dibenzo[*c*,*e*][1,2]dithiin (**3**) and its higher homologues **4**–**7** in high yields. The chemical structures were unambiguously validated by NMR, mass spectrometry, and single‐crystal X‐ray analyses, and UV/Vis absorption spectra revealed lowering of the optical energy gaps upon the structural extension. Moreover, dibenzo[1,2]dithiin **6** demonstrated maximum specific capacity of 118 mAh g^−1^ as cathode materials in lithium‐ion batteries, highlighting the value of such dibenzo[1,2]dithiin homologues for battery technologies. Furthermore, the current synthetic approach can potentially be extended to higher homologues with end‐to‐end overlap, leading to helical enantiomers^11^ of dibenzo[1,2]dithiin homologues.

## Experimental Section

**Crystallographic data**: Deposition numbers  1910696 (**4**), 1910697 (**5**), 1910698 (**6**), 1910690 (**8**), 1910694 (**9**), 1910695 (**10**), 1910691 (**17**), 1910692 (**19**), 1910693 (**23**) contain(s) the supplementary crystallographic data for this paper. These data are provided free of charge by the joint Cambridge Crystallographic Data Centre and Fachinformationszentrum Karlsruhe Access Structures service. Please see the Supporting Information for more details.

## Conflict of interest

The authors declare no conflict of interest.

## Supporting information

As a service to our authors and readers, this journal provides supporting information supplied by the authors. Such materials are peer reviewed and may be re‐organized for online delivery, but are not copy‐edited or typeset. Technical support issues arising from supporting information (other than missing files) should be addressed to the authors.

Supplementary
