# Supplementary material for: Oligophenyls with Multiple Disulfide Bridges as Higher Homologues of Dibenzo[c,e][1,2]dithiin: Synthesis and Application in Lithium‐Ion Batteries
Source: Chemistry. 2020 Jun 5;26(36):8007–11. doi: 10.1002/chem.202000728 (PMC7383724; doi:10.1002/chem.202000728)
Supplement: Supplementary file 1 — Supplementary [file CHEM-26-8007-s001.pdf]

Chemistry–A European Journal

Supporting Information

Oligophenyls with Multiple Disulfide Bridges as Higher Homologues of Dibenzo[*c*,*e*][1,2]dithiin: Synthesis and Application in Lithium-Ion Batteries

Christoph Sonnenschein,[a] Christopher P. Ender,[a] Faxing Wang,[b] Dieter Schollmeyer,[c] Xinliang Feng,[b] Akimitsu Narita,[a] and Klaus Müllen*[a]

Table of Contents

1. Synthesis....2

2. Crystal structures 12

3. Explanation of the MALDI-ToF spectra of thiomethyl oligophenylenes **8**-**12**....21

4. HPLC-studies of **6** and **6 crude** 22

5. Mechanism of the formation of benzothiophenes 23

6. Electrochemical measurements 24

7. NMR-Spectra 26

8. References....43

1. Synthesis

General. Thin layer chromatography (TLC) was performed on silica gel 60 F_{254} plates, spots were detected by fluorescence quenching under UV light at 254 nm. Column chromatography was performed on silica gel 60 (0.040-0.063 mm). All oxygen sensitive experimental procedures were carried out in head-gun dried glassware under the atmosphere of argon. Additional degassing was performed by argon bubbling. All nuclear magnetic resonance (NMR) spectra were recorded on a Bruker Avance III 300, 500 or 700 spectrometer at 25 °C in CD_2Cl_2, $C_2D_2Cl_4$ or THF-d_8. ^{1}H NMR (300, 500, 700 MHz) spectra were referenced to the solvent residual proton signal (CD_2Cl_2, δ_H = 5.32 ppm; $C_2D_2Cl_4$, δ_H = 6.0; THF-d_8, δ_H = 3.58 ppm). ^{13}C NMR (75, 125, 176 MHz) spectra with total decoupling of protons were referenced to the solvent (CD_2Cl_2, δ_c = 54.00 ppm; $C_2D_2Cl_4$, δ_C = 73.78; THF-d_8, δ_c = 67.57 ppm). UV-Vis spectra were recorded with a Perkin-Elmer Lambda 900 spectrophotometer in a 10.00 mm quartz cell at ambient temperature. Excitation coefficient ε is given in L (mol cm)$^{-1}$. Field desorption (FD-MS) spectra were obtained with a VG-Instruments ZAB-2SE-FPD at 8 V in dichloromethane. High resolution (HR-MS) spectra were obtained with a Matrix Assisted Laser Desorption Ionization technique and detected by Time of Flight (MALDI-ToF: YNAPT G2 Si high resolution time-of-flight mass spectrometer produced by WatersCorp). The samples were measured from solutions in tetrahydrofuran (THF) or by mechanically mixing solids with matrix: tetracyanochinodimethan (TCNQ). The treatment with a neodymium-doped yttrium aluminium garnet (Nd-YAG) LASER at 335 nm with 5-15 ns pulses resulted in the HR-MS spectra. Elemental analyses were performed using Heraeus Vario El after drying the respective sample in high vacuum (10^{-3} mbar). Melting points (MP) were recorded with Melting Point B-545 (Büchi) in steps of 2 °C/min. Crystal structures were obtained by a diffractometer (type IPDS2T of STOE & Cie GmbH) with monochromatic graphit-Mo-K_α-beam and solved with Direct methods. The crystals visualized the anisotropic displacement parameters at 50% level using mercury. High Pressure Liquid Chromatography (HPLC) was performed on a reversed phase C_{18}-column (l = 100 mm, d_{inner} = 4.6 mm, particle size = 3.5 µm; Agilent Eclipse RP18 Plus) with a water:THF gradient, going from 1:1 up to 100% THF using a flow-rate of 1 mL/min at 20 °C. The whole system was from Agilent Technologies 1200 Series with a Diode Array Detector (DAD 1290 Infinity II).

Materials. All starting materials, reagents and (dry) solvents were obtained from commercial suppliers and used without further purification.

2,2'-Bis(methylthio)-1,1'-biphenyl (8)

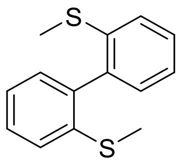

8

A suspension of 1-brom-2-(methylthio)benzene (**13**) (0.50 g, 2.5 mmol, 1.0 eq), and 2-(methylthio)benzene boronic acid (**16**) (0.58 g, 3.5 mmol, 1.4 eq) in toluene (25 mL), ethanol (5 mL) and an aqueous solution of Na_2CO_3 (1 M, 7 mL) was degassed by argon bubbling for 15 min. Then $Pd(PPh_3)_4$ (113 mg, 0.10 mmol, 0.04 eq) was added and the mixture was stirred for 36 h under reflux. The aqueous layer was extracted with 3 x 25 mL CH_2Cl_2. The organic layers were combined, dried over Na_2SO_4 and filtrated. The solvents were removed in vacuo and the residue was purified by column chromatography (silica, eluent: hexane:ethyl acetate = 5:1) yielding a colourless powder of **8** (0.56, 92%). ^{1}H NMR (DMSO-d_8, 500 MHz, δ in ppm): 2.33 (s, 6H), 7.07 (d, J_3 = 7.7 Hz, 2H), 7.19 (t, J_3 = 7.6 Hz, 2H), 7.33 (d, J_3 = 7.7 Hz, 2H), 7.38 (t, J_3 = 7.6 Hz, 2H). ^{13}C NMR (DMSO-d$_8$, 125 MHz, δ in ppm): 15.0, 125.2, 125.8, 128.5, 128.9, 131.4, 137.4. MP: 205 °C. MS (FD, positive) *m/z* calcd for $C_{14}H_{14}S_2$ $[M]^+$ 246.1, found 247.0; Anal Calcd for $C_{14}H_{14}S_2$: C, 68.3; H, 5.7. Found: C, 68.4; H, 5.8. All spectroscopic data was in agreement with the literature.[1]

Dibenzo[*c*,*e*][1,2]dithiin (3)

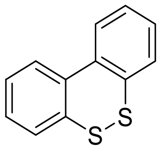

3

Under an atmosphere of argon pieces of sodium (46 mg, 2.0 mmol, 5 eq) and naphthalene (312 mg, 2.44 mmol, 6 eq) were stirred in dry THF (4 mL). After around 1 h the resulting dark green coloured reagent was added dropwise to a solution of 2,2'-bis(methylthio)-1,1'-biphenyl (**8**) (0.10 g, 0.41 mmol) in dry THF (4 mL) and the reaction mixture was stirred for 12 h at room temperature. Subsequently, degassed methanol (3 mL) and then degassed water (5 mL) were added. Naphthalene was removed by extraction with degassed CH_2Cl_2 (2 x 10 mL). Next, degassed hydrochloric acid (2 M, 10 mL) was added and the resulting thiol was extracted with degassed CH_2Cl_2 (4 x 25 mL). The combined organic layers were dried with Na_2SO_4, and the solvent was removed after filtration. The crude product was then diluted in 100 mL THF and borate buffer (5 mL, 1 mM, pH = 8.1) was added and pH was adjusted to 8.5 with a 2 M KOH solution. The mixture was then exposed to the air and stirred for 2 h. The organic layer was separated, dried with Na_2SO_4 and the solvent was removed in vacuo. Purification by column chromatography (silica, eluent: hexane:ethyl acetate = 25:1) yielded **3** in quantitative yield as yellow powder. ^{1}H NMR (CD_2Cl_2, 300 MHz, δ in ppm): 7.22 (dt, J_3 = 7.6 Hz, 2H), 7.32 (dt, J_3 = 7.6 Hz, 2H), 7.43 (dd, J_3 = 7.7 Hz, 2H), 7.64 (dd, J_3 = 7.7 Hz, 2H), 8.04 (s, 1H); H,H-NOESY (7.79, 8.04). ^{13}C NMR (CD_2Cl_2, 75 MHz, δ in ppm): 126.0, 127.9, 128.2, 129.9, 131.6; MS (FD, positive) *m/z* 216.0 (calcd for $C_{18}H_{10}S_4$ $[M]^+$), 216.0 (found). Anal Calcd for $C_{12}H_8S_2$: C, 66.6; H, 3.7. Found: C, 66.8; H, 3.8.

All spectroscopic data was in agreement with the literature.[2]

2,2'',4',6'-Tetrakis(methylthio)-1,1':3',1''-terphenyl (9)

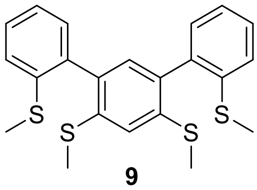

9

A suspension of **15**[3] (0.20 g, 0.61 mmol), **16** (0.37 g, 1.5 mmol, 2.4 eq) in dry THF (6 mL) and 1.2 mL 2 M Na_2CO_3 was degassed by argon bubbling for 20 min. Then $Pd(PPh_3)_4$ (28 µg, 24 mmol, 0.04 eq) was added and the mixture was stirred for 36 h under reflux. After separating the phases, the water layer was extracted with 3 x 20 ml CH_2Cl_2. The organic layers were combined, dried over Na_2SO_4 and filtrated. The solvents were removed and the residue was purified by column chromatography (silica, hexane:ethyl acetate = 5:1) yielding a colourless powder of **9** (1.1 g, 87%). MP: 138 - 139 °C. ^{1}H NMR (CD_2Cl_2, 500 MHz, δ in ppm) 2.42 (s, 6H), 2.49 (s, 6H), 7.68-6.58 (m, 10H). ^{13}C NMR (CD_2Cl_2, 125 MHz, δ in ppm) 15.6, 15.8, 121.3, 121.5, 124.4, 125.0, 125.4, 128.5, 130.2, 130.3, 131.7, 131.8, 135.1, 135.2, 138.0, 138.3, 138.3, 138.4, 138.6. HR-MS (MALDI-ToF, positive) *m/z* 414.0642 (calculated for $C_{22}H_{22}S_4$ $[M]^+$), 414.0641 (found). Anal Calcd for $C_{22}H_{22}S_4$: C, 63.7; H, 5.4. Found: C, 64.0; H, 5.3.

[1,1':3',1''-Terphenyl]-2,2'',4',6'-bis(dithiin) (4)

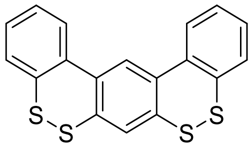

4

Under an atmosphere of argon pieces of sodium (55.4 mg, 2.41 mmol, 10 eq) and naphthalene (386 mg, 3.01 mmol, 12.5 eq) were stirred in dry THF (10 mL). After around 1 h the resulting dark green coloured reagent was added dropwise to a solution of 2,2'',4',6'-tetrakis(methylthio)-1,1':3',1''-terphenyl (**9**) (0.10 g, 0.24 mmol) in dry THF (14 mL) and the reaction mixture was stirred for 12 h at room temperature. Subsequently, degassed methanol (5 mL) and then degassed water (6 mL) were added. Naphthalene was removed by extraction with degassed CH_2Cl_2 (2 x 15 mL). Next, degassed hydrochloric acid (2 M, 20 mL) was added and the resulting thiol was extracted with degassed CH_2Cl_2 (4 x 30 mL). The combined organic layers were dried with Na_2SO_4, and the solvent was removed after filtration. The crude product was then diluted in 150 mL THF and borate buffer (5 mL, 1 mM, pH = 8.1) was added and pH was adjusted to 8.5 with a 2 M KOH solution. The mixture was then exposed to the air and stirred for 2 h. The organic layer was separated, dried with Na_2SO_4 and the solvent was removed in vacuo. Purification by column chromatography (silica, eluent: hexane:ethyl acetate = 25:1) yielded **4** as yellow powder (0.24 g, 98%). ^{1}H NMR (CD_2Cl_2, 500 MHz, δ in ppm): 7.35 (t, J_3 = 7.6 Hz, 2H), 7.44 (t, J_3 = 7.6 Hz, 2H), 7.56 (d, J_3 = 7.7 Hz, 2H), 7.68 (s, 1H), 7.79 (d, J_3 = 7.7 Hz, 2H), 8.04 (s, 1H); H,H-NOESY (7.79, 8.04). ^{13}C NMR (CD_2Cl_2, 125 MHz, δ in ppm): 127.0, 127.8, 128.4, 128.5, 128.9, 129.1, 135.6, 136.1, 137.4, 138.0. MS (MALDI-ToF, positive) *m/z* calcd for $C_{18}H_{10}S_4$ $[M]^+$ 353.97, found 353.93. Anal Calcd for $C_{18}H_{10}S_4$: C, 61.0; H, 2.8. Found: C, 61.5; H, 2.5.

2,2',2'',5'-Tetrakis(methylthio)-[1,1':4',1''-terphenyl] (**10**)

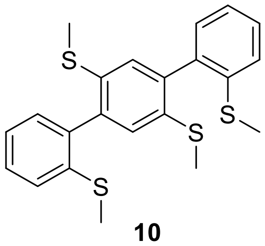

10

A suspension of **20**[4] (0.10 g, 0.30 mmol), and 2-(methylthio)benzene boronic acid (**16**) (0.18 g, 0.73 mmol, 2.4 eq) and 7 mL 1 M Na_2CO_3 in dry toluene (5 mL) and ethanol (1 mL) was degassed by argon bubbling for 15 min. Then $Pd(PPh_3)_4$ (14 mg, 12 µmol, 0.04 eq) was added and the mixture was stirred for 36 h under reflux. The aqueous layer was extracted with 3 x 15 mL of CH_2Cl_2. The organic layers were combined, dried over Na_2SO_4 and filtrated. The solvents were removed in vacuo and the residue was purified by column chromatography (silica, eluent: hexane:ethyl acetate = 5:1) yielding a colourless powder of **10** (113 mg, 89%). MP: 190 - 191 °C. ^{1}H NMR (THF-d_8, 500 MHz, δ in ppm) 2.24 (s, 3H), 2.26 (s, 3H), 2.32 (s, 3H), 3.34 (s, 3H), 7.38 - 7.10 (m, 10H). ^{13}C NMR (THF-d_8, 126 MHz, δ in ppm) 13.1, 13.6, 122.3, 123.8, 124.1, 126.1, 126.7, 128.3, 132.8, 136.6, 137.4, 137.8. FD-MS *m/z* 414.1 (calculated); 414.1 (found). HR-MS (MALDI-ToF, positive) *m/z* 367.0649 (calculated for $C_{21}H_{20}S_3$ [M-SMe]$^+$), 367.0628 (found) Fragmentation could not be suppressed. Anal Calcd for $C_{22}H_{22}S_4$: C, 63.7; H, 5.4. Found: C, 63.8; H, 5.3.

[1,1':4',1''-Terphenyl]-2, 2',2'', 5'-*bis*(dithiin) (**5**)

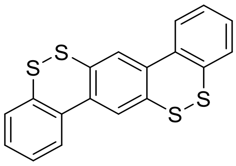

5

Under an atmosphere of argon pieces of sodium (55.4 mg, 2.41 mmol, 10 eq) and naphthalene (386 mg, 3.01 mmol, 12.5 eq) were stirred in dry THF (10 mL). After around 1 h the resulting dark green coloured reagent was added dropwise to a solution of 2,2',2'',5'-tetrakis(methylthio)-[1,1':4',1''-terphenyl] (**10**) (0.10 g, 0.24 mmol) in dry THF (14 mL) and stirred for 12 h at room temperature. Subsequently, degassed methanol (5 mL) and then degassed water (6 ml) were added. Naphthalene was removed by extraction with degassed CH_2Cl_2 (2 x 15 mL). Next, degassed hydrochloric acid (2 M, 20 mL) was added and the resulting thiol was extracted with degassed CH_2Cl_2 (4 x 30 mL). The combined organic layers were dried with Na_2SO_4, and the solvent was removed after filtration. The crude product was then diluted in 150 mL of THF and borate buffer (5 mL, 1 mM, pH = 8.1) was added and pH was adjusted to 8.5 with a 2 M KOH solution. The mixture was then exposed to the air and stirred for 2 h. The organic layer was separated, dried with Na_2SO_4 and the solvent was removed in vacuo. Purification by column chromatography (silica, eluent: hexane:ethyl acetate = 25:1) yielded **4** as yellow powder (0.23 g, 97%). MP: 222 °C. ^{1}H NMR (CD_2Cl_2, 500 MHz, δ in ppm): 7.90 (s, 2H), 7.79 (dd, *J* = 7.8, 1.4 Hz, 2H), 7.59 (dd, *J* = 7.8, 1.4 Hz, 2H), 7.47 (td, *J* = 7.6, 1.4 Hz, 2H), 7.39 (td, *J* = 7.6, 1.5 Hz, 2H). H,H-NOESY (7.90,7.79). ^{13}C NMR (CD_2Cl_2, 125 MHz, δ in ppm): 127.9, 128.6, 128.8, 129.1, 135.9, 136.4, 136.8, 137.2. HR-MS (MALDI-ToF, positive) *m/z* 353.9663 (calculated for $C_{18}H_{10}S_4$ [M]$^+$), 353.9661 (found). Anal Calcd for $C_{18}H_{10}S_4$: C, 61.0; H, 2.8. Found: C, 61.2; H, 2.6.

2,2''',4',4'',6',6''-Hexakis(methylthio)-[1,1':3',1'':3'',1'''-quaterphenyl] (11)

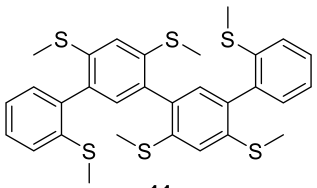

11

A suspension of **19** (0.50 g, 1.0 mmol), **16** (0.66 g, 2.6 mmol, 2.6 eq) in dry THF (10 mL) and 2 mL 2 M Na_2CO_3 was degassed by argon bubbling for 20 min. Then $Pd(PPh_3)_4$ (46.6 mg, 40.3 µmol, 0.04 eq) was added and the mixture was stirred for 36 h under reflux. After separating the phases, the water layer was extracted with 3 x 20 ml CH_2Cl_2. The organic layers were combined, dried over Na_2SO_4 and filtrated. The solvents were removed and the residue was purified by column chromatography (silica, hexane:ethyl acetate = 5:1) yielding a colourless powder of **11** (0.46 g, 78%). MP: 164 - 165 °C. ^{1}H NMR (CD_2Cl_2, 500 MHz, δ in ppm) 2.26 (m, 3H), 2.38 (s, 3H), 2.44 (s, 3H), 2.47 (s, 6H), 2.50 (s, 6H), 7.65 - 6.80 (m, 12H). ^{13}C NMR (CD_2Cl_2, 125 MHz, δ in ppm) 15.5, 15.7, 15.9, 117.1, 122.0, 124.5, 125.1, 128.8, 130.1, 133.6, 136.6, 137.1, 138.5, 138.6, 139.8. FD-MS *m/z* 582.1 (calculated); 582.0 (found). HR-MS (MALDI-ToF, positive) *m/z* 535.0706 (calculated for $C_{29}H_{27}S_5$ $[M\text{-}HSMe]^+$), 535.0689 (found), Fragmentation could not be suppressed. Anal Calcd for $C_{30}H_{30}S_6$: C, 61.8; H, 5.2. Found: C, 61.8; H, 5.0.

[1,1':3',1'':3'',1'''-Quaterphenyl]-2,2''',4',4'',6',6''-*tris*(dithiin) (6)

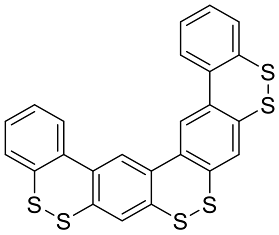

6

Under an atmosphere of argon pieces of sodium (118 mg, 5.15 mmol, 15 eq) and naphthalene (792 mg, 6.18 mmol, 18 eq) were stirred in dry THF (20 mL). After around 1 h the resulting dark green coloured reagent was added dropwise to a solution of 2,2''',4',4'',6',6''-Hexakis(methylthio)-[1,1':3',1'':3'',1'''-quaterphenyl] (**11**) (0.20 g, 0.32 mmol) in dry THF (44 mL) and stirred for 12 h at room temperature. Subsequently, degassed methanol (15 ml) and then degassed water (20 ml) were added. Naphthalene was removed by extraction with degassed CH_2Cl_2 (2 x 20 mL). Next, degassed hydrochloric acid (2 M, 20 mL) was added and the resulting thiol was extracted with degassed CH_2Cl_2 (4 x 40 mL). The combined organic layers were dried with Na_2SO_4 , and the solvent was removed after filtration. The crude product was then diluted in 200 mL THF and borate buffer (10 mL, 1 mM, pH = 8.1) was added and pH was adjusted to 8.5 with a 2 M KOH solution. The mixture was then exposed to the air and stirred for 2 h. The organic layer was separated, dried with Na_2SO_4 and the solvent was removed in vacuo. Purification by column chromatography (silica, eluent: hexane:ethyl acetate = 4:1) yielded **6** as yellow powder (0.15 g, 90%). MP: 225 °C. ^{1}H NMR (CD_2Cl_2, 500 MHz, δ in ppm): 7.36-7.32 (m, 2H), 7.29-7.26 (m, 2H). 7.48 (d, *J* = 7.7 Hz, 2H), 7.63 (s, 2H), 7.69 (d, *J* = 7.8, 2H), 7.99 (s, 2H). ^{13}C NMR (CD_2Cl_2, 125 MHz, δ in ppm): 126.9, 127.8, 128.5, 128.7, 129.0, 129.2, 137.3, 137.5, 138.2. FD-MS *m/z* 491.9 (calculated); 492.0 (found). MS (MALDI-ToF, positive) *m/z* 492.9336 (calculated for $C_{24}H_{13}S_6$ $[M+H]^+$), 492.9261 (found). Anal Calcd for $C_{24}H_{12}S_6$: C, 58.5; H, 2.4. Found: C, 58.5; H, 2.4.

2,2',2'',2''',5',5''-Hexakis(methylthio)-[1,1':4',1'':4'',1'''-quaterphenyl] (12)

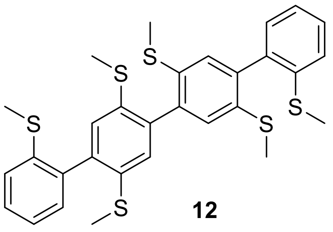

12

A suspension of **23** (0.10 g, 0.15 mmol), **14** (0.11 g, 0.66 mmol, 3.3 eq) and 1 mL 1 M Na_2CO_3 in dry toluene (5 mL) and ethanol (0.8 mL) was degassed by argon bubbling for 15 min. Then $Pd(PPh_3)_4$ (21 mg, 18 µmol, 0.06 eq) was added and the mixture was stirred for 36 h under reflux. The aqueous layer was extracted with 3 x 10 mL CH_2Cl_2. The organic layers were combined, dried over Na_2SO_4 and filtrated. The solvents were removed in vacuo and the residue was purified by column chromatography (silica, eluent: hexane:ethyl acetate = 3:1) yielding a colourless powder of **12** (0.10 g, 85%). MP: 285 - 286 °C. ^{1}H NMR (THF-d_8, 700 MHz, δ in ppm): 2.26-2.38 (m, 18H), 7.07-7.39 (m, 12H). ^{13}C NMR (CD_2Cl_2, 176 MHz, δ in ppm): 15.5, 15.6, 16.0, 124.5, 125.1, 127.3, 127.3, 127.4, 128.6, 130.3, 134.4, 138.3. FD-MS *m/z* 582.1 (calculated); 582.1 (found). HR-MS (MALDI-ToF, positive) *m/z* 535.0713 (calculated for $C_{29}H_{27}S_5$ [M-SMe]$^+$), 535.0720 (found); Fragmentation could not be suppressed.

[1,1':4',1'':4'',1'''-Quaterphenyl]-2,2',2'',2''',5',5''-tris(dithiin) (7)

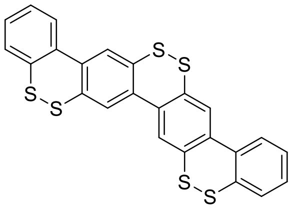

7

Under an atmosphere of argon pieces of sodium (11 mg, 0.52 mmol, 15 eq) and naphthalene (79 mg, 0.62 mmol, 18 eq) were stirred in dry THF (2 mL). After around 1 h the resulting dark green coloured reagent was added dropwise to a solution of 2,2',2'',2''',5',5''-Hexakis(methylthio)-[1,1':4',1'':4'',1'''-quaterphenyl] (**12**) (0.02 g, 0.03 mmol) in dry THF (5 mL) and stirred for 12 h at room temperature. Subsequently, degassed methanol (4 ml) and then degassed water (6 ml) were added. Naphthalene was removed by extraction with degassed CH_2Cl_2 (2 x 20 mL). Next, degassed hydrochloric acid (2 M, 20 mL) was added and the resulting thiol was extracted with degassed CH_2Cl_2 (4 x 40 mL). The combined organic layers were dried with Na_2SO_4, and the solvent was removed after filtration. The crude product was then diluted in 200 mL THF and borate buffer (10 mL, 1 mM, pH = 8.1) was added and pH was adjusted to 8.5 with a 2 M KOH solution. The mixture was then exposed to the air and stirred for 2 h. The organic layer was separated, dried with Na_2SO_4 and the solvent was removed in vacuo. Purification by column chromatography (silica, eluent: hexane:ethyl acetate = 4:1) yielded **6** as yellow powder (10 mg, 60%). ^{1}H NMR (CD_2Cl_2, 500 MHz, δ in ppm): 7.08 (s, 2H), 7.40-7.50 (m, 8H), 7.93 (s, 2H). ^{13}C-NMR spectrum of **7** could not be properly measured due to the poor solubility of **7**. HR-MS (MALDI-ToF, positive) *m/z* 459.9541 (calculated for $C_{24}H_{12}S_5$ [M-S]$^+$), 459.9545 (found), *m/z* 427.9821 (calculated for $C_{24}H_{12}S_4$ [M-S_2]$^+$), 427.9815 (found), Fragmentation could not be suppressed.

2-{5-Bromo-2,4-bis(methylthio)benzene}-4,4,5,5-tetramethyl-1,3,2-dioxaborolane (17)

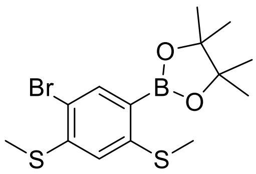

17

To a solution of **15** (1.0 g, 3.1 mmol) in dry THF (30 mL) was added a solution of *n*-butyllithium (2.3 mL, 1.2 eq, 1.6 M in hexane) dropwise at –98 °C under argon. After stirring for 2 h at this temperature, 2-isopropoxy-4,4,5,5-tetramethyl-1,3,2-dioxaborolane (0.85 g, 4.6 mmol, 1.5 eq) was added. The solution was then warmed up to room temperature stirred for another 1 h, followed by addition of 1 M HCl (20 mL). After stirring for 10 min, the reaction mixture was extracted with 3 x 40 mL CH_2Cl_2. The organic layers were combined, dried over Na_2SO_4 and evaporated. The residue was purified by column chromatography (silica, eluent: hexane:ethyl acetate = 5:1) and recrystallization with hexane providing the desired product **17** as a colourless crystal (1.0 g, 90% yield). MP: 118 - 119 °C. ^{1}H NMR (CD_2Cl_2, 300 MHz, δ in ppm): 1.33 (s, 12H), 2.46 (s, 3H), 2.51 (s, 3H), 6.88 (s, 1H), 7.75 (s, 1H). ^{13}C NMR (CD_2Cl_2, 75 MHz, δ in ppm): 15.6, 16.4, 25.2, 84.2, 116.8, 121.1, 136.8, 139.7, 143.7, 146.0. **FD-MS** *m/z* 374.0 (calculated); 374.1 (found).

4-Bromo-6-iodo-1,3-bis(methylthio)benzene (18)

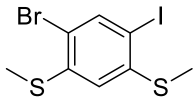

18

To a solution of **15** (1.5 g, 4.6 mmol) in dry THF (46 mL) was added a solution of *n*-butyllithium (3.4 mL, 1.2 eq, 1.6 M in hexane) dropwise at –-98 °C under argon. After stirring for 2 h at this temperature, iodine (1.7 g, 6.9 mmol, 1.5 eq) in 5 mL dry THF was added until the colour of the solution remained violet. The solution was then warmed up to room temperature and stirred for another hour h, followed by addition of distilled water (20 mL). After extraction with 3 x 40 mL CH_2Cl_2, the organic layers were combined, dried over Na_2SO_4 and evaporated. The residue was purified by column chromatography (silica, eluent: hexane:ethyl acetate = 5:1) providing the desired product **11** as a colourless powder (1.6 g, 92% yield). MP: 114-115 °C. ^{1}H NMR (CD_2Cl_2, 300 MHz, δ in ppm): 2.49 (s, 3H), 2.50 (s, 3H), 6.83 (s, 1H), 7.87 (s, 1H). ^{13}C NMR (CD_2Cl_2, 75 MHz, δ in ppm): 16.1, 17.6, 121.8, 122.2, 128.9, 129.5, 141.8. FD-MS *m/z* 373.8 (calculated); 373.8 (found). Anal Calcd for $C_8H_8S_2BrI$: C, 25.6; H, 2.2. Found: C, 25.9; H, 2.3.

5,5'-Dibromo-2,2',4,4'-tetrakis(methylthio)-1,1'-biphenyl (19)

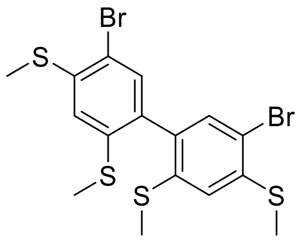

19

A suspension of **18** (1.0 g, 2.7 mmol), **17** (1.1 g, 2.9 mmol, 1.1 eq) and 13 mL 1 M K_2CO_3 in dry toluene (25 mL) was degassed by argon bubbling for 40 min. Then $Pd(PPh_3)_2Cl_2$ (47 mg, 66 mmol, 0.03 eq) was added and the mixture was stirred for 16 h under reflux. The aqueous layer was extracted with 3 x 25 mL CH_2Cl_2. The organic layers were combined, dried over Na_2SO_4 and filtrated. The solvents were removed in vacuo and the residue was purified by column chromatography (silica, eluent: hexane:ethyl acetate = 20:1 to 4:1) yielding a colourless powder of **19** (0.82 g, 62%). MP: 194 - 195 °C. ^{1}H NMR (CD_2Cl_2, 300 MHz, δ in ppm): 2.40 (s, 6H), 2.55 (s, 6H), 7.01 (s, 2H), 7.27 (s, 2H). ^{13}C NMR (CD_2Cl_2, 75 MHz, δ in ppm): 16.0, 16.3, 122.1, 122.3, 134.0, 139.0, 140.8, 147.4. FD-MS *m/z* 495.9 (calculated); 495.9 (found). Anal Calcd for $C_{16}H_{16}S_4Br_2$: C, 38.7; H, 3.3. Found: C, 38.4; H, 3.1.

2-Bromo-5-iodo-1,4-*bis*(methylthio)benzene (21)

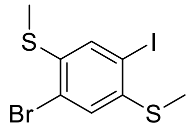

21

To a solution of **20** (1.0 g, 3.1 mmol) in dry THF (30 mL) was added a solution of *n*-butyllithium (2.3 mL, 1.2 eq, 1.6 M in hexane) dropwise at –98 °C under argon. After stirring for 2 h at this temperature, iodine (0.93 g, 3.6 mmol, 1.2 eq in 3.7 mL THF) was added. The solution was then warmed up to room temperature stirred for another 1 h. After stirring for 10 min, the reaction mixture was extracted with 3 x 30 mL CH_2Cl_2. The organic layers were combined, dried over $MgSO_4$ and evaporated. The residue was purified by column chromatography (silica, eluent: hexane:ethyl acetate = 10:1) and washed with aceton providing the desired product **21** as a colourless powder (0.9 g, 79% yield). MP: 124-125 °C. ^{1}H NMR (CD_2Cl_2, 300 MHz, δ in ppm): 7.42 (s, 1H), 7.14 (s, 1H), 2.38 (s, 6H). ^{13}C NMR (CD_2Cl_2, 75 MHz, δ in ppm): 16.4, 17.6, 121.9, 123.1, 128.9, 129.5, 136.0. FD-MS *m/z* 373.8 (calculated); 374.0 (found). Anal Calcd for $C_8H_8S_2BrI$: C, 25.6; H, 2.2. Found: C, 25.2; H, 2.3.

4-Bromo-2,5-bis(methylthio)benzene boronic acid (22)

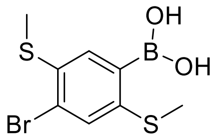

22

To a solution of **20** (1.0 g, 3.1 mmol) in dry THF (30 mL) was added a solution of *n*-butyllithium (2.3 mL, 1.2 eq, 1.6 M in hexane) dropwise at –98 °C under argon. After stirring for 2 h at this temperature, trimethylborate (0.44 g, 4.3 mmol, 1.4 eq) was added. The solution was then warmed up to room temperature stirred for another 1 h, followed by addition of 3 M HCl (20 mL). After stirring for 20 min, the reaction mixture was extracted with 4 x 40 mL CH_2Cl_2. The organic layers were combined, dried over $MgSO_4$ and evaporated. The residue was purified by recrystallization with hexane yielding a colourless powder of **22** (0.84 g, 94%). ^{1}H NMR (THF-d_8, 300 MHz, δ in ppm): 2.43 (s, 3H), 2.45 (s, 3H), 5.77 (s, 2 H), 7.34 (s, 1H), 7.55 (s, 1H). ^{13}C NMR (THF-d_8, 75 MHz, δ in ppm) 15.9, 19.3, 123.1, 130.2, 132.9, 134.4. Anal Calcd for $C_7H_{10}S_2BrBO_2$: C, 32.8; H, 3.4. Found: C, 33.1; H, 3.2.

4,4'-Dibromo-2,2',5,5'-tetrakis(methylthio)-[1,1'-biphenyl] (23)

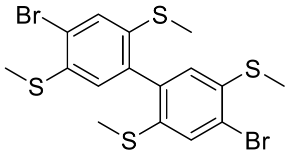

23

A suspension of **21** (1.0 g, 2.7 mmol), **22** (0.86 g, 2.9 mmol, 1.1 eq) and 6 mL 2 M Na_2CO_3 in dry toluene (25 mL) and ethanol (3 ml) was degassed by argon bubbling for 40 min. Then $Pd(PPh_3)_4$ (12 mg, 0.1 mmol, 0.04 eq) was added and the mixture was stirred for 36 h at 60 °C. The aqueous layer was extracted with 3 x 20 mL CH_2Cl_2. The organic layers were combined, dried over Na_2SO_4 and filtrated. The solvents were removed in vacuo and the residue was purified by column chromatography (silica, eluent: hexane:ethyl acetate = 20:1 to 4:1) yielding a colourless powder of **23** (0.82 g, 82%). MP: 189 - 190 °C. ^{1}H NMR (CD_2Cl_2, 300 MHz, δ in ppm) 2.30 (s, 6H), 2.32 (s, 6H), 6.84 (s, 2H), 7.37 (s, 2H). ^{13}C NMR (CD_2Cl_2, 75 MHz, δ in ppm) 16.3, 16.5, 122.3, 127.5, 130.0, 136.3, 136.4, 138.1. FD-MS *m/z* 495.9 (calculated); 495.9 (found). Anal Calcd for $C_{16}H_{16}S_4Br_2$: C, 38.7; H, 3.3. Found: C, 38.5; H, 3.2.

Dibenzo[b,b']thieno[3,2-f:4,5-f']-bis[1]benzothiophene (24)

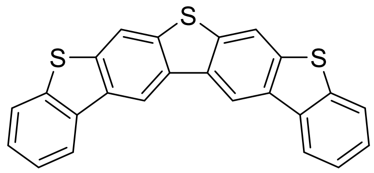

24

Under an atmosphere of argon **6** (10 mg, 20 µmol) was refluxed with triphenyl arsenide (32 mg, 0.11 mmol, 5.2 eq) in 2 mL DMPU (1,3-Dimethyl-1,3-diazinan-2-one) for 4 d in a pressure vessel. Afterwards the mixture was poured into water and extracted with CH_2Cl_2 (5 x 10 ml). The combined layers were washed with water (5 x 20 ml), dried with $MgSO_4$ and the solvent was removed. **24** was purified by column chromatography (silica, hexane:ethyl acetate = 4:1) yielding a colourless powder with 56% (4.5 mg). MP: 157 °C. ^{1}H NMR (CD_2Cl_2, 300 MHz, δ in ppm): 8.86 (s, 2H), 8.29-8.23 (m, 4H), 8.21 (s, 2H), 7.83-7.77 (m, 4H). ^{13}C NMR (CD_2Cl_2, 75 MHz, δ in ppm): 114.3, 116.5, 121.8, 123.3, 125.0, 127.9, 128.8, 129.0, 133.2, 135.6, 139.1, 139.7. FD-MS *m/z* 396.0 (calculated); 396.3 (found).

2. Crystal structures

[1,1':3',1''-Terphenyl]-2,2'',4',6'-bis(dithiin) **4** [CCDC-Nr. 1910696]

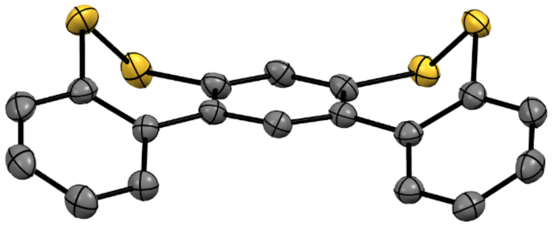

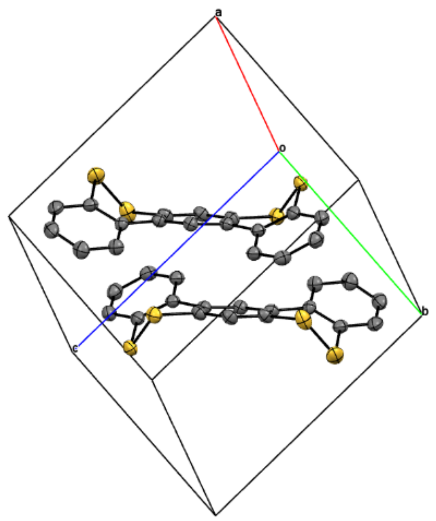

Chemical formula	$C_{18}H_{14}S_4$	
Formula mass	358.55 $gmol^{-1}$	
Space group	P -1	
Crystal system	triclinic	
Absorption	μ = 0.62 mm^{-1} correction with 6 crystal faces	
Transmission	T_{min} = 0.82, T_{max}= 0.96	
Crystal size	0.07 x 0.10 x 0.49 mm³ yellow plate	
Lattice parameter	a = 7.6606(8)Å	α = 93.626(8)°
(calculated from	b = 10.0464(10)Å	ß = 110.037(7)°
7933 reflections with	c = 10.5199(10)Å	γ = 94.906(8)°
2.8°< θ < 28.2°)	V = 754.12(13)$Å^3$	z = 2 F(000) = 364.0
Temperature	-80°C	
Density	$d_{x\text{-}ray}$ = 1.56 gcm^{-3}	
Number of reflections		
total/unique/obs.(I>2σ(I))	7207/3650/2565	
Number of refined parameters	199	
Final wR2/R1(obs)/R1(all)/	0.2310/0.0631/0.0911	
Max./min peak height	1.126/-0.692	

[1':4',1''-Terphenyl]-2, 2',2'', 5'-*bis*(dithiin) **5** [CCDC-Nr. 1910697]

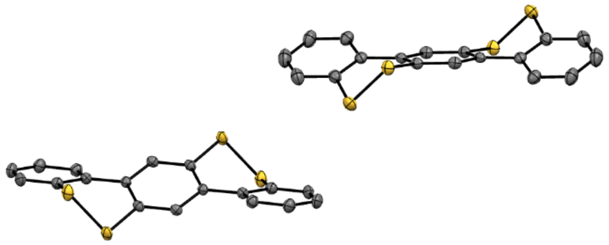

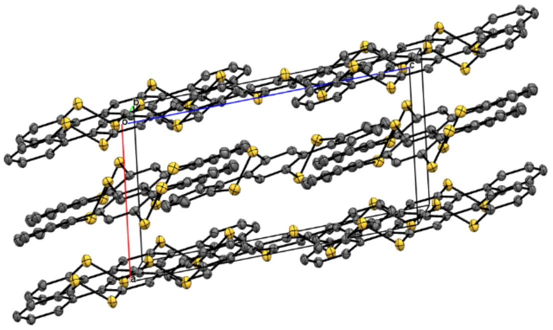

Chemical formula	$C_{18}H_{10}S_4$		
Formula mass	354.54 $gmol^{-1}$		
Space group	P 2_1/c		
Crystal system	monoclinic		
Absorption	μ = 0.63 mm^{-1} correction with 6 crystal faces		
Transmission	T_{min} = 0.8204, T_{max}= 0.9688		
Crystal size	0.04 x 0.08 x 0.56 mm^3 yellow needle		
Lattice parameter	a = 7.5278(4)Å		
(calculated from	b = 14.2425(9)Å	ß = 98.188(4)°	
6996 reflections with	c = 14.0432(6)Å		
2.05°< θ < 28.35°)	V =1490.29(13)$Å^3$	z = 4	F(000) = 728
Temperature	-80°C		
Density	$d_{x\text{-}ray}$ = 1.580 gcm^{-3}		
Number of reflections total/unique/obs.(I>2σ(I))	8175/3653/2615		
Number of refined parameters	199		
Final wR2/R1(obs)/R1(all)/	0.0865/0.0351/0.0616		
Max./min peak height	0.317/-0.312		

[1,1':3',1'':3'',1'''-Quaterphenyl]-2,2''',4',4'',6',6''-*tris*(dithiin) **6** [CCDC-Nr. 1910698]

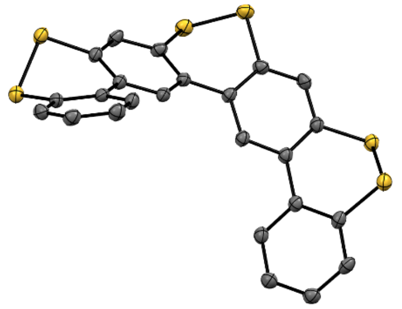

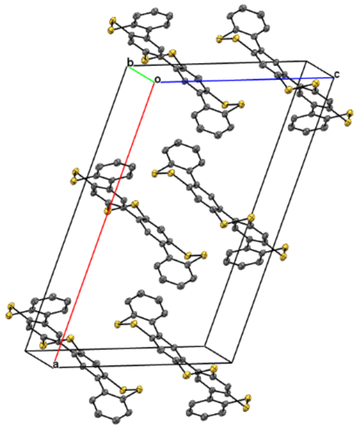

Chemical formula	$C_{24}H_{12}S_6$		
Formula mass	492.7 gmol^{-1}		
Space group	C 2/c		
Crystal system	monoclinic		
Absorption	μ = 0.69 mm^{-1}		
Transmission	T_{min} = 0.8688, T_{max}= 0.9839		
Crystal size	0.02 x 0.20 x 0.21 mm^3 bright yellow plate		
Lattice parameter	a = 21.843(3)Å		
(calculated from	b = 7.2454(5)Å	ß = 109.222(8)°	
6886 reflections with	c = 13.4221(14)Å		
3°< θ < 28.2°)	V = 2005.8(4)Å^3	z = 4	F(000) = 1008
Temperature	120K		
Density	$d_{x\text{-}ray}$ = 1.632 gcm^{-3}		
Number of reflections total/unique/obs.(I>2σ(I))	4979/2429/1677		
Number of refined parameters	137		
Final wR2/R1(obs)/R1(all)/	0.1396/0.0564/0.0965		
Max./min peak height	0.474/-0.423		

2,2'-Bis(methylthio)-1,1'-biphenyl **8** [CCDC-Nr. 1910690]

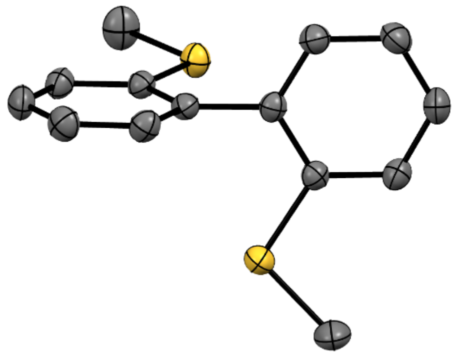

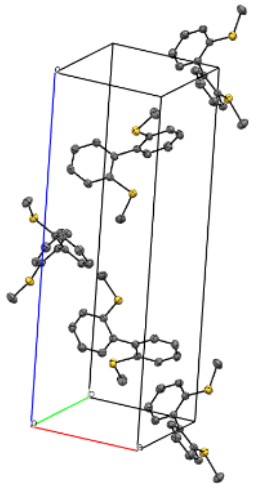

Chemical formula	$C_{14}H_{14}S_2$
Formula mass	246.37 $gmol^{-1}$
Space group	P 4_12_12
Crystal system	tetragonal
Absorption	μ = 0.401 mm^{-1}
Crystal size	0.21 x 0.31 x 0.41 mm^3 colourless block
Lattice parameter	a = 7.3668(10)Å
(calculated from	c = 22.677(5)Å
6600 reflections with	
2.9°< θ < 28.4°)	V = 1230.7(4)$Å^3$ z = 4 F(000) = 520
Temperature	-80°C
Density	$d_{x\text{-}ray}$ = 1.33 gcm^{-3}
Number of reflections total/unique/obs.(I>2σ(I))	3670/1510/1377
Number of refined parameters	99
Final wR2/R1(obs)/R1(all)/	0.0852/0.0329/0.0385
Flack parameter	-0.08(10)
Max./min peak height	0.247,-0.47

2,2'',4',6'-Tetrayltetrakis(methylthio)-[1,1':3',1''-terphenyl] 9 [CCDC-Nr. 1910694]

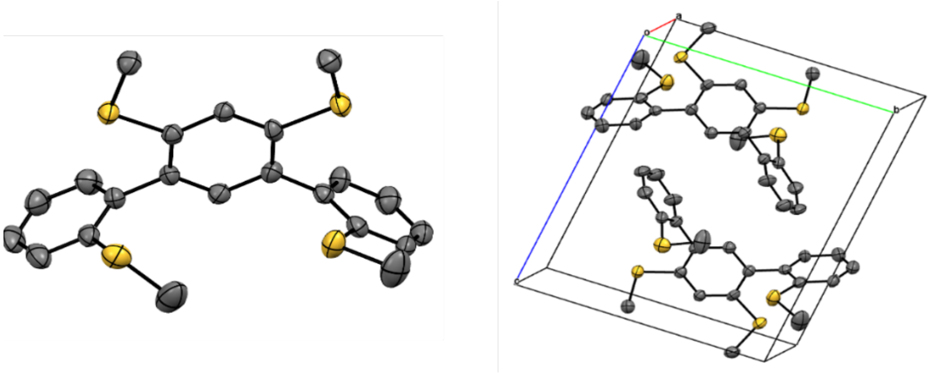

Chemical formula	$C_{22}H_{22}S_4$		
Formula mass	414.67 $gmol^{-1}$		
Space group	P -1		
Crystal system	triclinic		
Absorption	μ = 0.46 mm^{-1} correction with 6 crystal faces		
Transmission	T_{min} = 0.917, T_{max}= 0.986		
Crystal size	0.03 x 0.13 x 0.25 mm^3 colourless block		
Lattice parameter	a = 7.2677(7)Å	α = 97.824(7)°	
(calculated from	b = 11.3766(9)Å	β = 90.039(8)°	
5056 reflections with	c = 12.9309(13)Å	γ = 99.570(7)°	
2.2°< θ < 28.2°)	V = 1044.16(17)$Å^3$	z = 2	F(000) = 436.0
Temperature	-80°C		
Density	d_{x-ray} = 1.319 gcm^{-3}		
Number of reflections total/unique/obs.(I>2σ(I))	10222/5172/2609/		
Number of refined parameters	239		
Final wR2/R1(obs)/R1(all)/	0.2139/0.0667/0.1539		
Max./min peak height	0.500/-0.459		

2,2',2'',5'-Tetrakis(methylthio)-[1,1':4',1''-terphenyl] **10** [CCDC-Nr. 1910695]

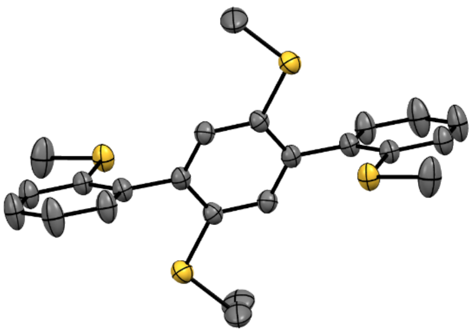

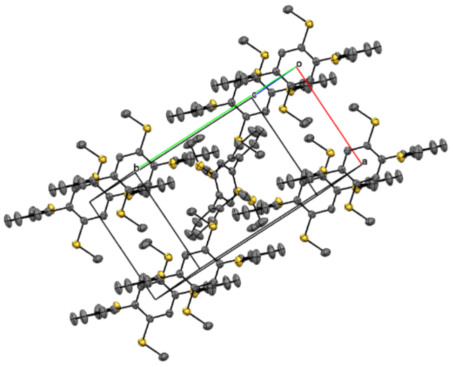

Chemical formula	$C_{22}H_{22}S_4$		
Formula mass	414.66 $gmol^{-1}$		
Space group	$P\ 2_1/n$		
Crystal system	monoclinic		
Absorption	μ = 0.46 mm^{-1}		
Crystal size	0.04 x 0.17 x 0.17 mm^3 colourless plate		
Lattice parameter	a = 8.0247(4)Å		
(calculated from	b = 14.3453(7)Å	ß = 91.789(5)	
8811 reflections with	c = 9.0848(5)Å		
2.6°< θ < 28.4°)	V = 1045.30(10)$Å^3$	z = 2	F(000) = 436.0
Temperature	-80°C		
Density	$d_{x\text{-}ray}$ = 1.317 gcm^{-3}		
Number of reflections total/unique/obs.(I>2σ(I))	5909/2584/2058		
Number of refined parameters	155		
Final wR2/R1(obs)/R1(all)/	0.1376/0.0518/0.0698		
Max./min peak height	1.064/-0.321		

2-(5-Brom-2,4-bis(methylthio)phenyl)-4,4,5,5-tetramethyl-1,3,2-dioxaborolan **17** [CCDC-Nr. 1910691]

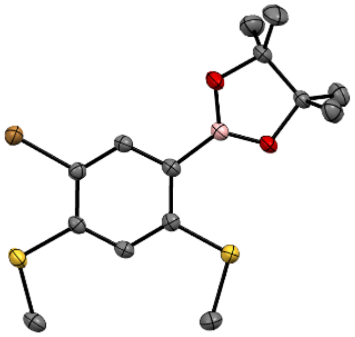

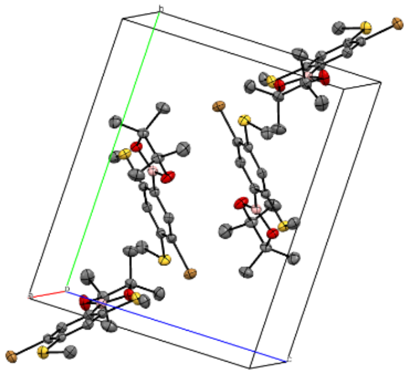

Chemical formula	$C_{14}H_{20}BBrO_2S_2$		
Formula mass	375.14 gmol^{-1}		
Space group	P 2_1/c		
Crystal system	monoclinic		
Absorption	µ = 2.775 mm^{-1} correction with 6 crystal faces		
Transmission	T_{min} = 0.3011, T_{max}= 0.5909		
Crystal size	0.2 x 0.48 x 0.51 mm³ colourless block		
Lattice parameter	a = 11.4919(4)Å		
(calculated from	b = 13.7191(4)Å	ß = 111.412(3)°	
12148 reflections with	c = 11.1064(4)Å		
2.2°< θ < 28.4°)	V = 1630.16(10)Å^3	z = 4	F(000) = 768
Temperature	-80°C		
Density	$d_{x\text{-ray}}$ = 1.529 gcm^{-3}		
Number of reflections total/unique/obs.(I>2σ(I))	8042/4016/3652		
Number of refined parameters	249		
Final wR2/R1(obs)/R1(all)/	0.0747/0.0298/0.0346		
Max./min peak height	0.443/-0.500		

2,2',4,4'-Tetrakis(methylthio)-5,5'-dibrom-[1,1'-biphenyl] **19** [CCDC-Nr. 1910692]

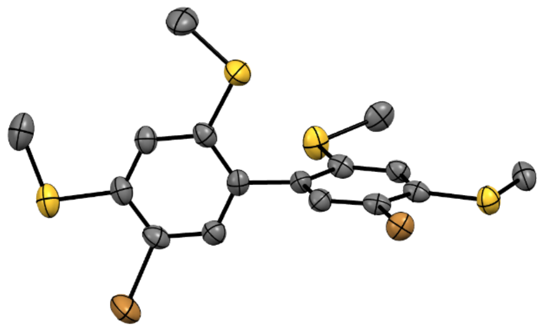

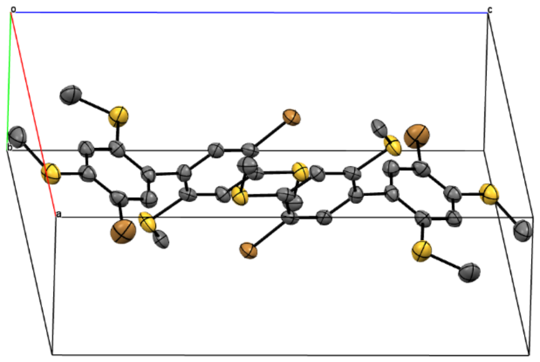

Chemical formula	$C_{16}H_{16}Br_2S_4$		
Formula mass	496.35 $gmol^{-1}$		
Space group	P -1		
Crystal system	triclinic		
Absorption	μ = 4.765 mm^{-1}		
Transmission	T_{min} = 0.2108, T_{max}= 0.6059 correction with 6 crystal faces		
Crystal size	0.11 x 0.16 x 0.43 mm^3 colourless block		
Lattice parameter	a = 7.3689(8)Å		α = 86.765(9)°
(calculated from	b = 8.4328(10)Å		β = 78.898(9)°
10079 reflections with	c = 16.4377(18)Å		γ = 69.207(8)°
2.6°< θ < 28.2°)	V = 936.97(19)$Å^3$	z = 2	F(000) = 492
Temperature	-80°C		
Density		d_{x-ray} = 1.759 gcm^{-3}	
Number of reflections total/unique/I>2σ(I)	8870/4550/2870		
Number of refined parameters	203		
Final wR2/R1(obs)/R1(all)/	0.1916/0.0638/0.1151		
Max./min peak height	0.867/-1.195		

2,2',4,4'-Tetrakis(methylthio)-5,5'-dibrom-[1,1'-biphenyl] **23** [CCDC-Nr. 1910693]

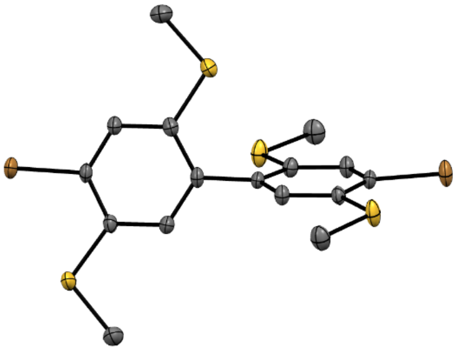

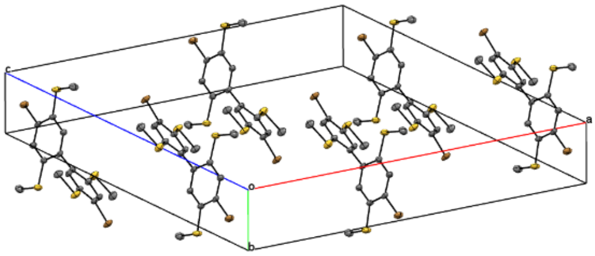

Chemical formula	$C_{16}H_{16}Br_2S_4$		
Formula mass	493.85 $gmol^{-1}$		
Space group	C 2/c		
Crystal system	monoclinic		
Absorption	μ = 4.97 mm^{-1} correction with 7 crystal faces		
Transmission	T_{min} = 0.2657, T_{max}= 0.5720		
Crystal size	0.13 x 0.13 x 0.28 mm^3 colourless block		
Lattice parameter	a = 22.0551(10)Å		
(calculated from	b = 6.4238(3)Å	ß = 135.015(3)°	
5837 reflections with	c = 17.9898(9)Å		
1.8°< θ < 28.4°)	V = 1801.76(15)$Å^3$	z = 4	F(000) = 984
Temperature	-80°C		
Density		$d_{x\text{-}ray}$ = 1.83 gcm^{-3}	
Number of reflections total/unique/I>2σ(I)	4409/2199/1820		
Number of refined parameters	102		
Final wR2/R1(obs)/R1(all)/	0.0846/0.0341/0.0462		
Max./min peak height	0.626/-0.351		

3. Explanation of the MALDI-ToF spectra of thiomethyl oligophenylenes **8-12**

Due to the fragile thiomethyl groups, MALDI-ToF spectra of oligophenylenes **8**-**12** did not show strong signals corresponding to their pristine structures while displaying more intense peaks from other species after removal of thiomethyl groups (see Fig. S1 and S2).

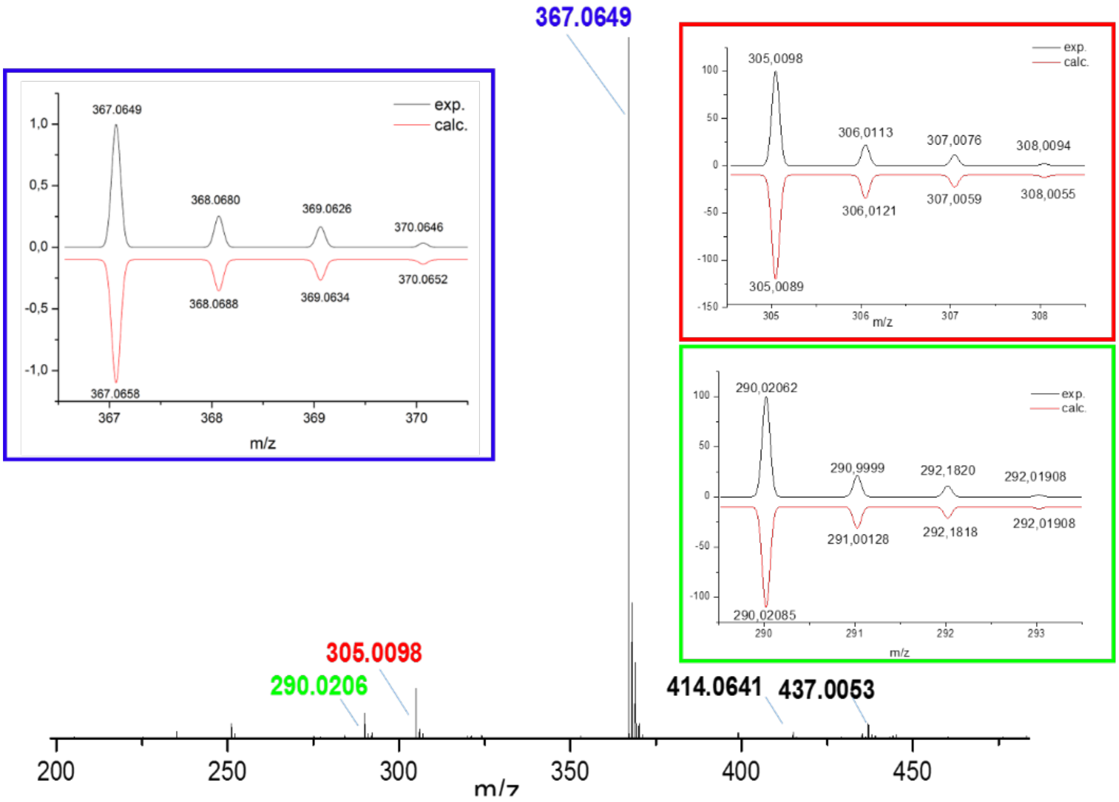

Fig. S1 MALDI-ToF spectra of thiomethylated oligophenylene **9**. Inset shows isotopic distributions of the different peaks, which can be assigned to **S1**, **S2** and **S3** that are generated during the measurements as displayed in Fig. S2. The signal at *m*/*z* = 437.0053 corresponds to $[M+Na]^+$.

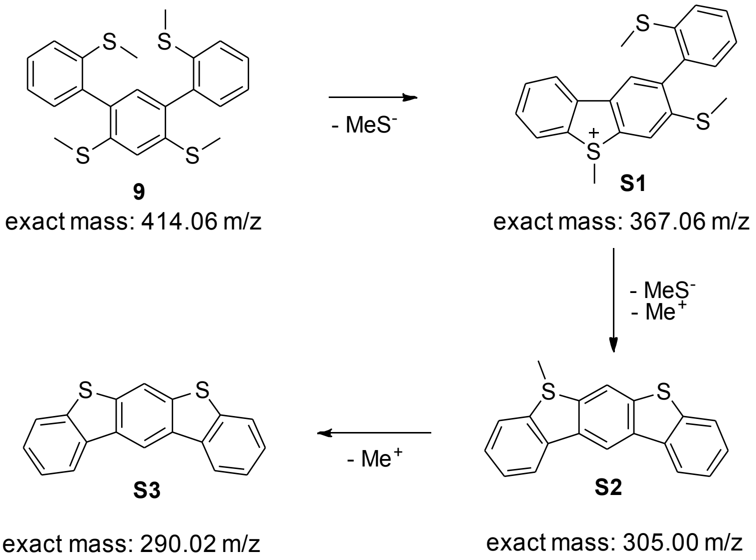

Fig. S2 Generation of **S1**, **S2** and **S3** during the MALDI-ToF measurement of **9**.

4. HPLC-studies of 6 and 6 crude

A crude product of **6** (**6-crude**) was analyzed by HPLC along with **6** purified by recrystallization and dibenzo[b,b']thieno[3,2-f:4,5-f']-bis[1]benzothiophene (**24**) as the completely desulfonated byproduct, which was separated synthesized (Fig. S 3). The HPLC chart of **6crude** revealed mainly four peaks: The one at 10.8 min could be assigned to **6** und the one at 8.7 min to **24**. This result proves that the benzothiophene moieties were formed during the reductive demethylation and oxidative [1,2]dithiin-ring formation starting from **11**. The other peaks in the HPLC charts of **6** can most probably be assigned to other byproducts with one or two benzothiophene moieties.

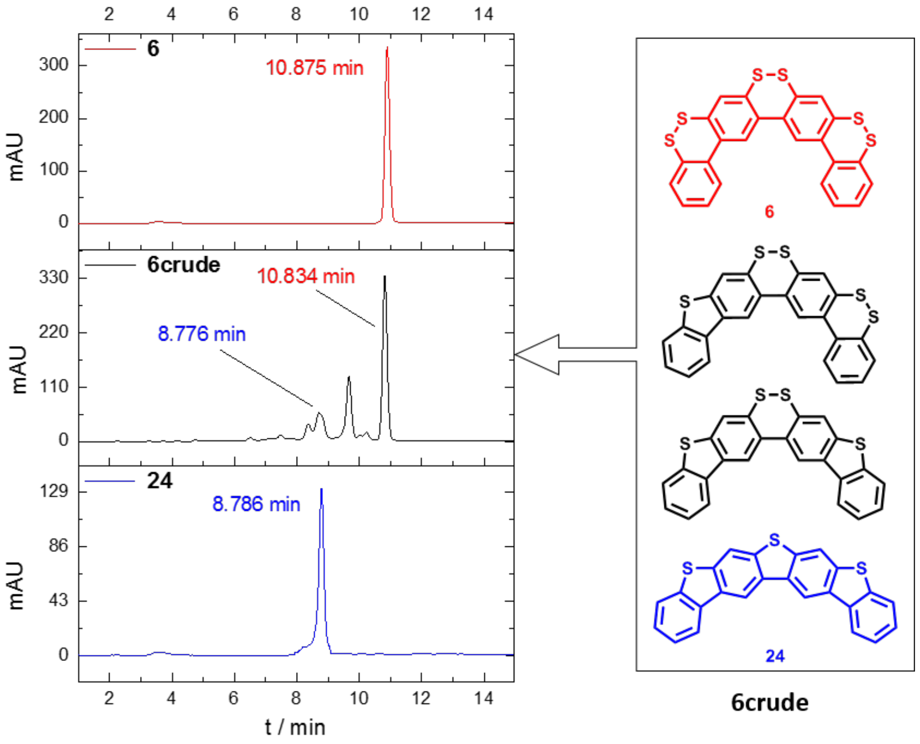

Fig. S3 HPLC of **6, 6crude** and dibenzo[*b*,*b*']thieno[3,2-f:4,5-f']-*bis*[1]benzothiophene (**24**) (1 ml/min, 20 °C, starting from 1:1 H_2O:THF and going by gradient to 100% THF).

5. Mechanism of the formation of benzothiophenes

In Fig. S 4 the proposed mechanism for the formation of the benzothiophene species is shown. During the reductive deprotection thiolates are formed. Sometimes opposite to formed thiolates are still methyl thiol groups at the neighboring phenyls. Therefore, as a nucleophile the thiolate substitutes the methyl thiol before its deprotection and forms a benzothiophene, while two neighbored thiolates remain and are oxidized in the following step to a disulfide, forming a [1,2]dithiin ring.

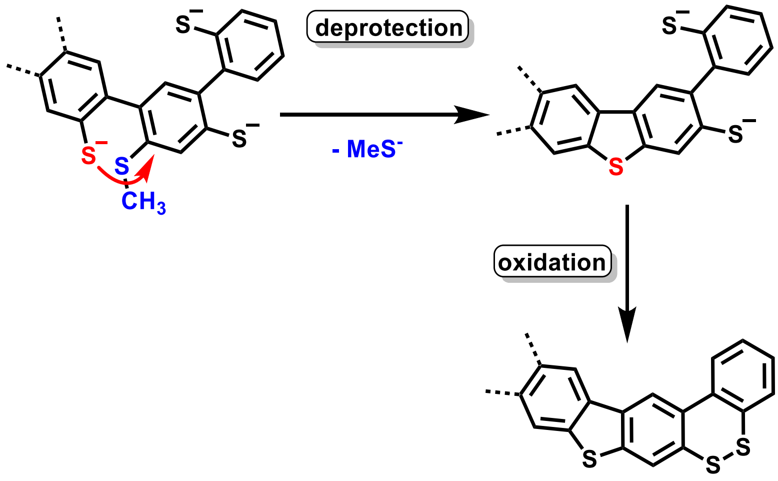

Fig. S 4 Proposed mechanism of the formation of benzothiophene species during the deprotection.

6. Electrochemical measurements

The working electrode was prepared by coating the N-methyl-2-pyrrolidone (NMP)-based slurry containing the **4** (or **6**), acetylene black and polyvinylidene difluoride (PVDF) in a weight ratio of 6:3:1 on aluminum foil using a doctor-blade technique. The coated foils were dried and punched into circular pieces (d =11mm). Cell assembly was carried out in an argon-filled glove box with the contents of oxygen and water below 0.1 ppm. The electrolyte used was 1 M $LiCF_3SO_3$ (LiTFSI) in a solvent mixture of 1,3-dioxolane (DOL)/1,2-dimethoxyethane (DME) (1:1 v/v). A lithium foil was used as the counter electrode and separated by a Celgard 2400 microporous film. The cyclic voltammetry was obtained at a scan rate of 1 mV s^{-1} on a CHI 660E electrochemical workstation (Chenhua Co., Ltd., Shanghai, China). The cells were galvanostatically charge/discharged at different current densities in the potential window of 1.5 to 3 V on a LAND electrochemical instrument (CT2001A). Based on these considerations, electrochemical behaviors of **4** and **6** for Li ion storage were evaluated using standard 2032-type coin cells with Li foil anode and 1M LiTFSI electrolyte.

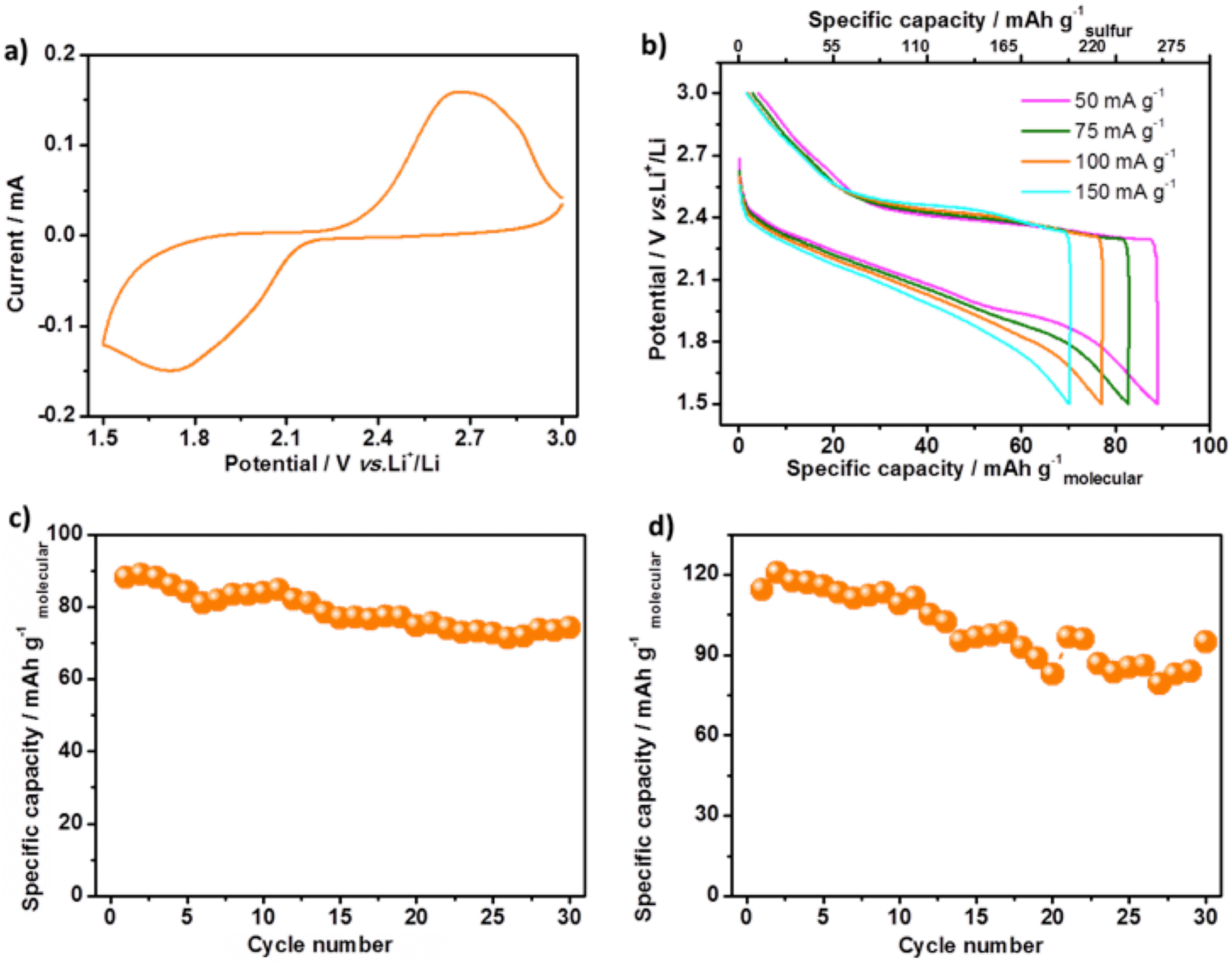

Fig. S5 a) Cyclic voltammogram (CV) curve of **4** and b) galvanostatic charge/discharge curves of **4.** The cycling behaviors of c) compound **4** and (d) compound **6** at 50 mA g^{-1}.

Finally, we have examined the potential of dibenzo[1,2]dithiins **4** and **6** for applications as electrode materials for Li ion storage. Especially, the structures of **4** and **6**, where the S–S bonds are fixed to a carbon-backbone with high sulfur-carbon ratios, might hinder the loss of intermediate lithium polysulfide species (Li_2S_x, $4 \leq x \leq 8$) caused by the so-called "shuttle" effect.[5] For pure sulphur electrode, the produced lithium polysulfide species

during the discharge process are highly soluble in the electrolyte, which can freely diffuse to the anode and irreversibly react with Li metal. Such lithium polysulfide shuttle effect may cause the serious active material loss and rapid battery capacity decay. One promising method to alleviating the shuttle effect was to combine the S-S bond to the carbon backbone with an intramolecular redox reaction between the S-S bonds and the Li ions. The fact that a reductive opening of the sulfur-sulfur bonds remains an intramolecular process is thus advantageous for the use as the cathode material for Li ion storage.

The electrochemical properties of the dibenzo[1,2]dithiins 4 and 6 electrodes for Li ion storage were evaluated by cyclic voltammograms (CV) and galvanostatic charge/discharge tests in the voltage range of 1.5-3V. Electrodes prepared with **4** and **6** both showed very symmetrical anodic and cathodic redox peaks in the CV curves which indicated excellent reversibility of the redox reaction with Li-ions (Fig. S5a). The electrode with **4** exhibited an initial charge and discharge capacity of 84 and 89 $mAhg^{-1}$ at a current density of 50 mA g^{-1} (Fig. S 5b). On the other hand, the electrode with **6** demonstrated specific capacities of 118, 110, 104 and 84 mAh g^{-1} at 50, 75, 100 and 150 mA g^{-1}, respectively, based on the mass of **6**. The higher specific capacity of **6** compared to **4** results from the higher sulfur content per molecule. The cycling stabilities of the electrodes were studied at 50 mA g^{-1} for 30 cycles. The specific capacity of **6** was stabilized at around 74 mA g^{-1}, corresponding to 91% capacity retentions and a small capacity fading of only 0.3% per cycle (Fig. S5c). Similarly, a stable cycling behavior was demonstrated in the **6** electrode with 84% capacity retention after 30 cycles (Fig. S5d). In addition, the charged electrode of **6** readily powered 33 commercial red light-emitting diodes (LED, 1.7–2.3 V) in parallel connection (Fig. S6), suggesting a great potential application of **6** molecular for Li ion storage.

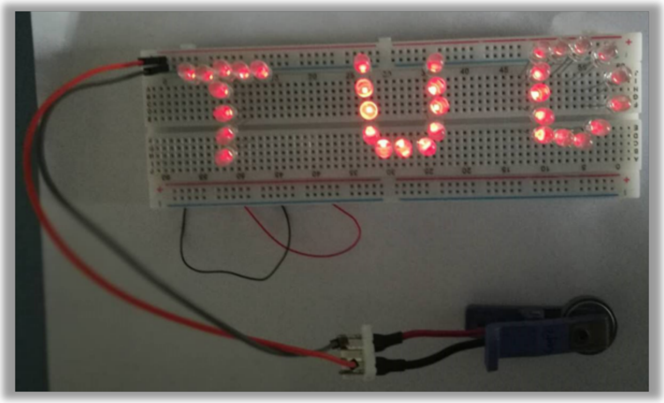

Fig. S6 An optical photograph showing lighted red LEDs powered by a charged lithium-ion battery based on dibenzo[1,2]dithiin **6** as the electrode

7. NMR-Spectra

^{1}H NMR (300 MHz, CD_2Cl_2) of dibenzo[*c*,*e*][1,2]dithiin (3)

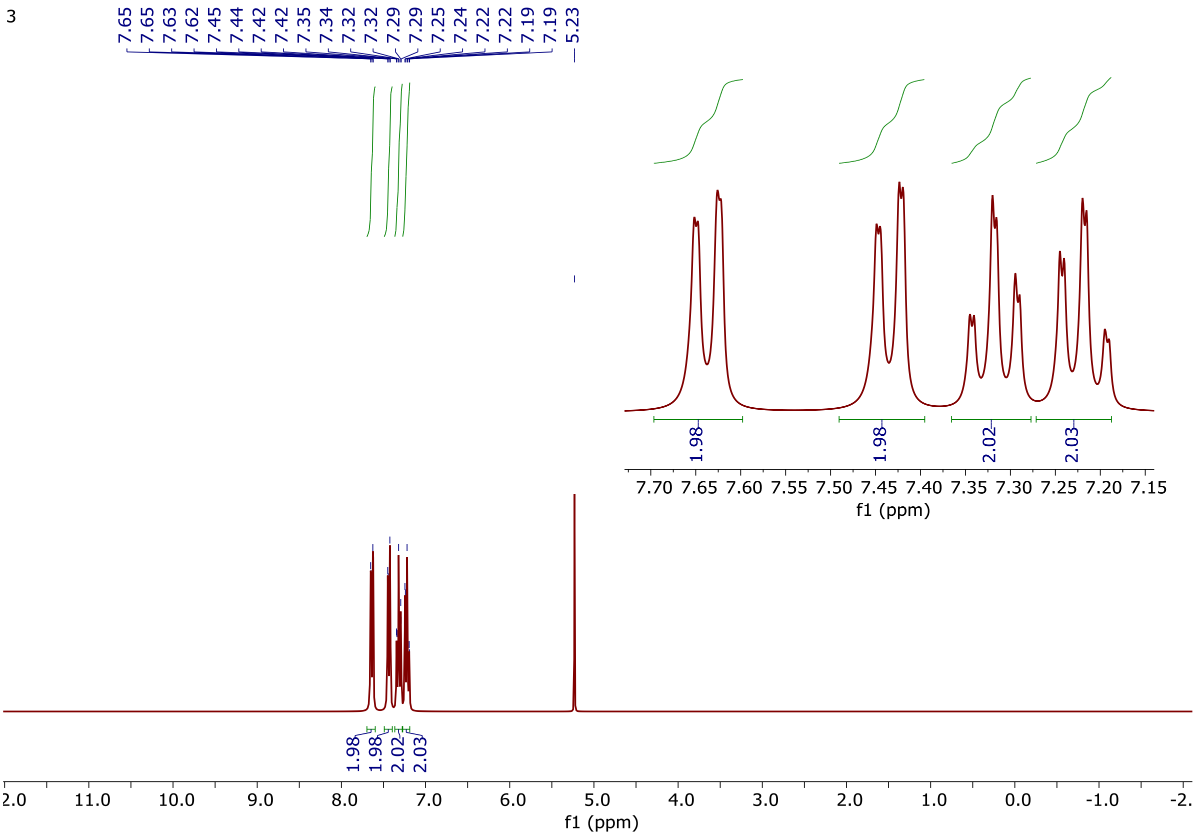

^{1}H NMR (300 MHz, CD_2Cl_2) of 2,2'-bis(methylthio)-1,1'-biphenyl (8)

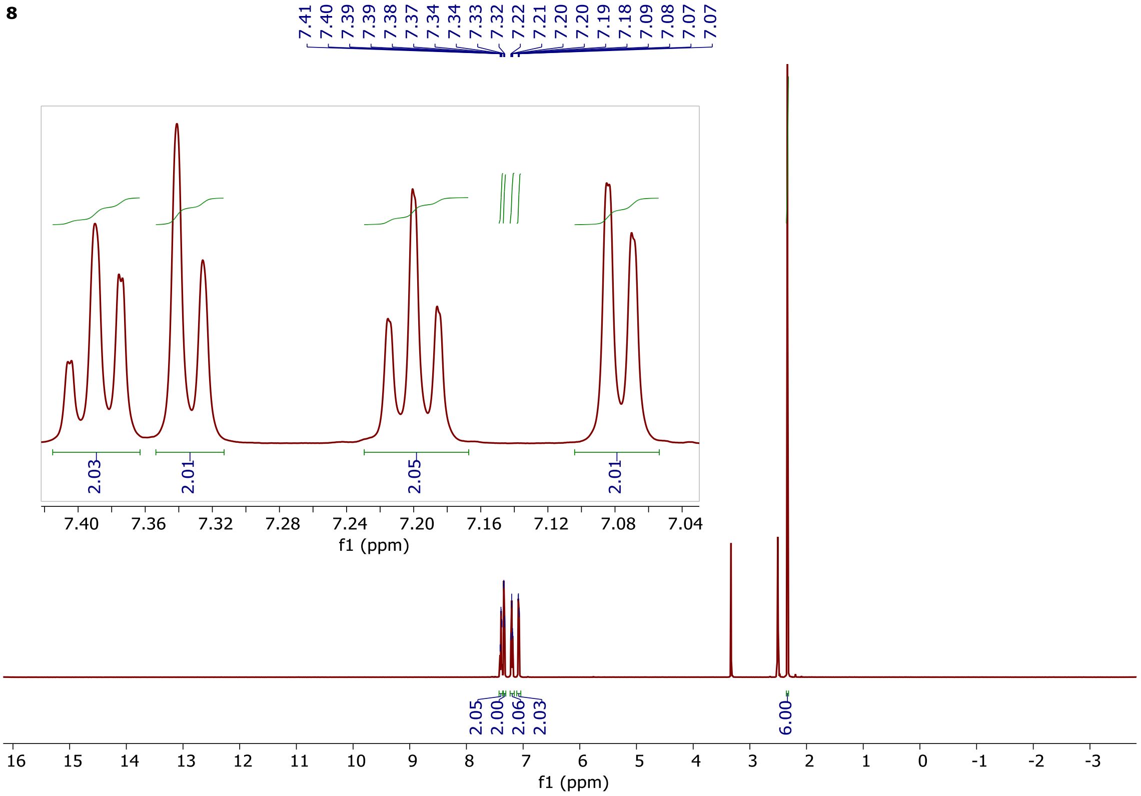

^{1}H NMR (500 MHz, CD_2Cl_2) of [1,1':3',1''-terphenyl]-2,2'',4',6'-bis(dithiin) (**4**)

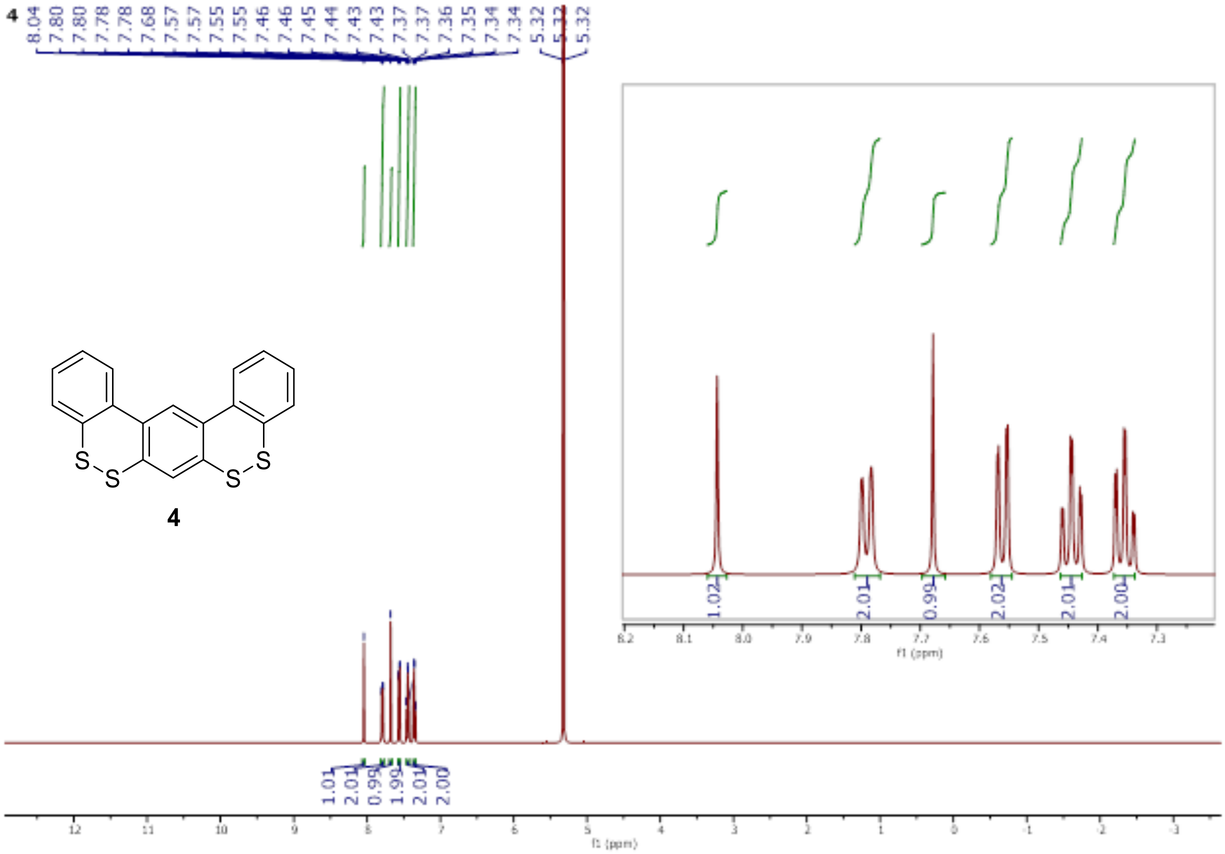

^{13}C NMR (500 MHz, CD_2Cl_2) of [1,1':3',1''-terphenyl]-2,2'',4',6'-bis(dithiin) (**4**)

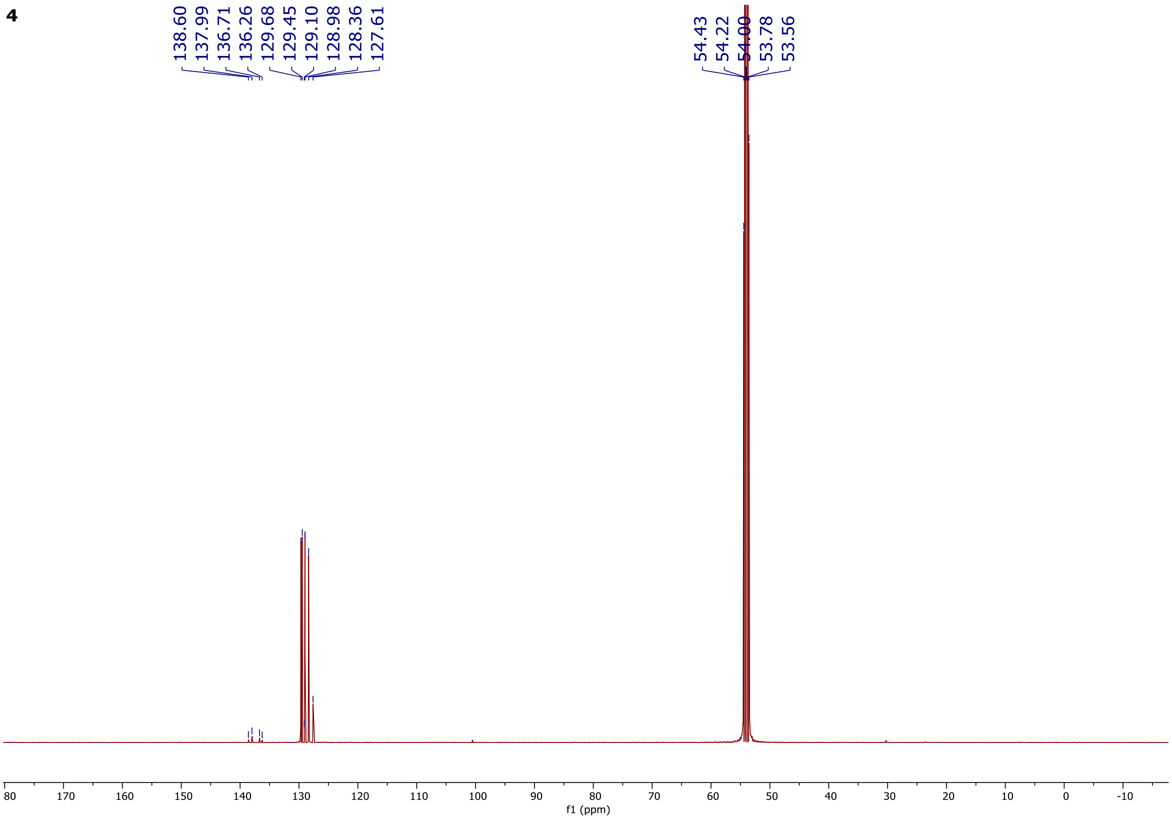

NOESY-NMR (500 MHz, CD_2Cl_2) of [1,1':3',1''-terphenyl]-2,2'',4',6'-bis(dithiin) (**4**)

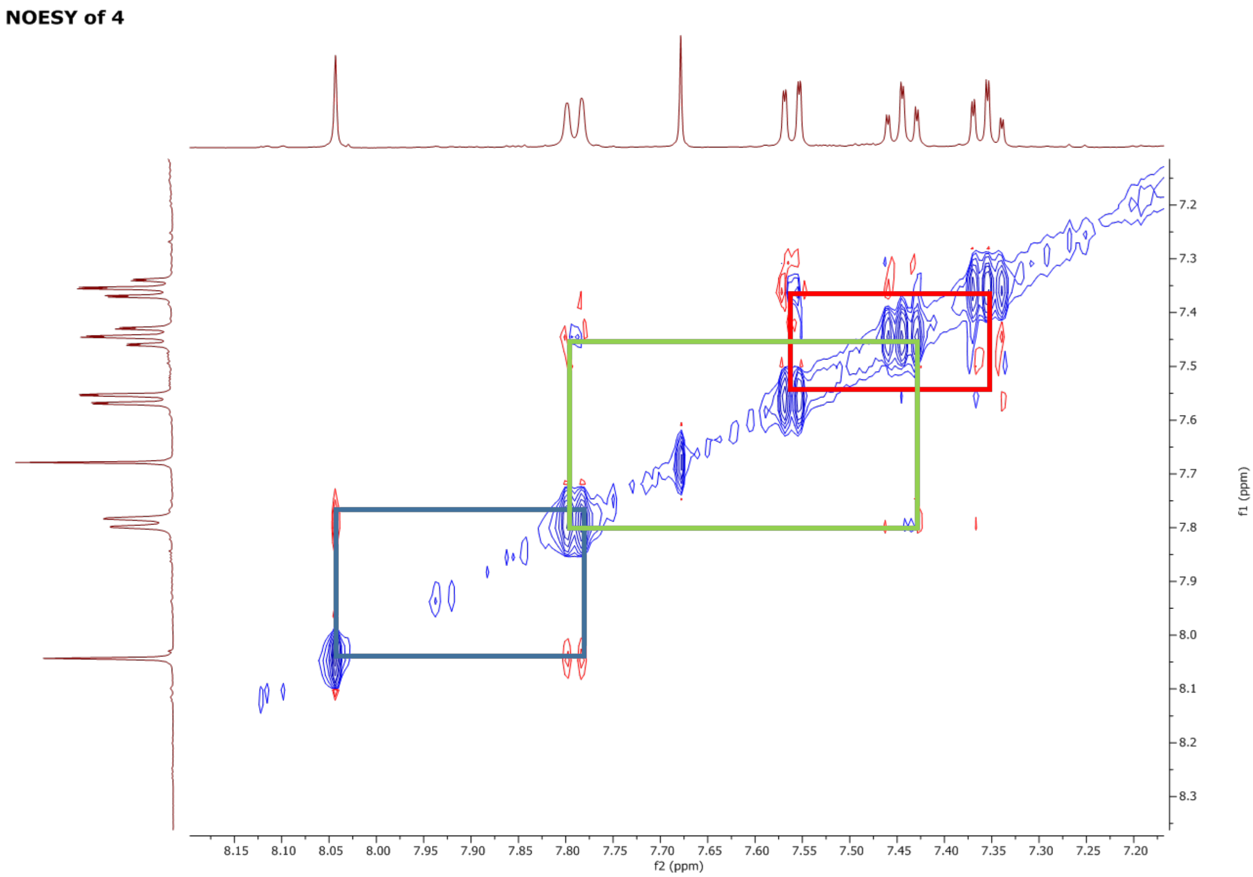

^{1}H NMR (500 MHz, CD_2Cl_2) of [1,1':4',1''-terphenyl]-2, 2',2'', 5'-bis(dithiin) (**5**)

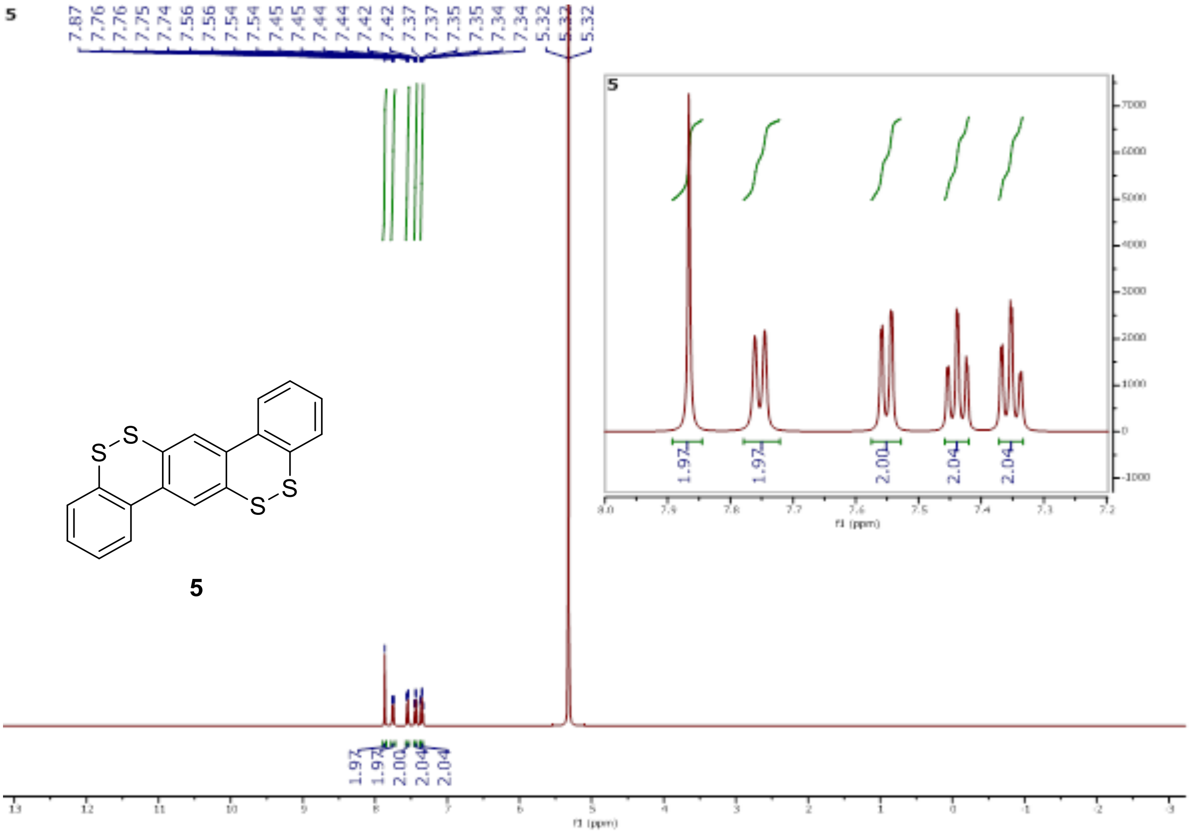

^{13}C NMR (126 MHz, CD_2Cl_2) of [1,1':4',1''-terphenyl]-2, 2',2'', 5'-bis(dithiin) (**5**)

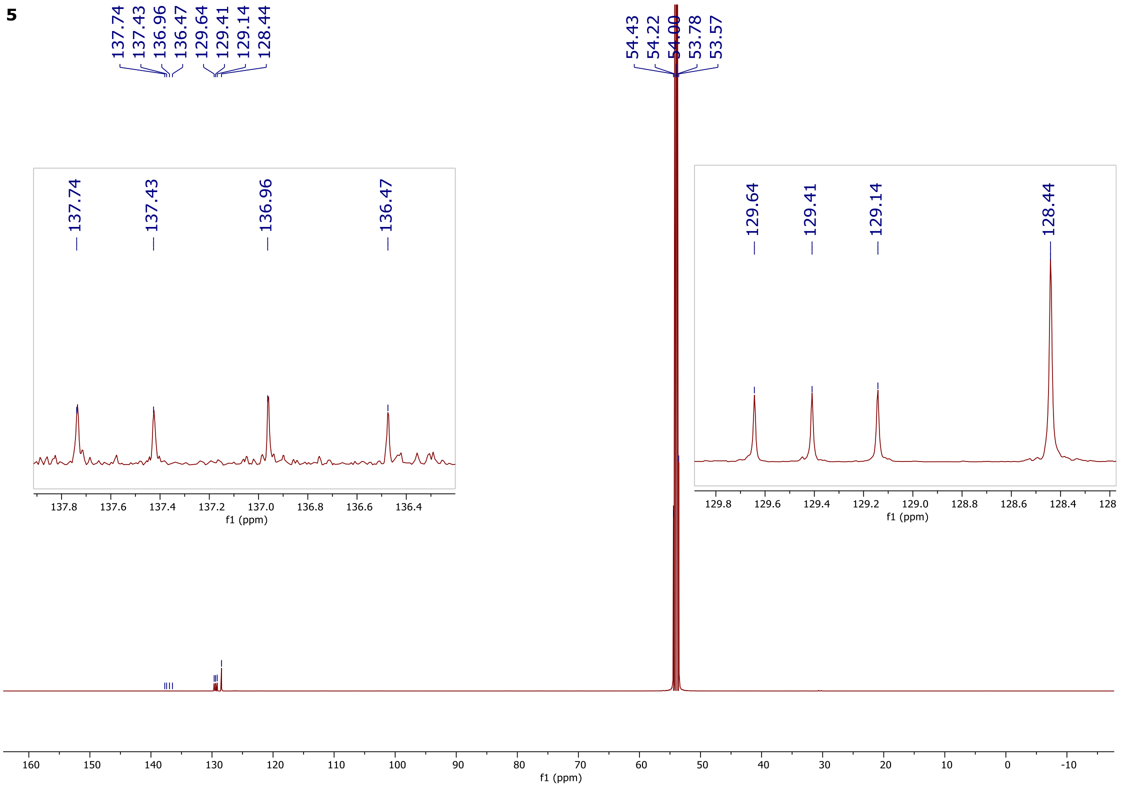

^{1}H NMR (500 MHz, CD_2Cl_2) of [1,1':3',1'':3'',1'''-quaterphenyl]-2,2''',4',4'',6',6''-tris(dithiin) (**6**)

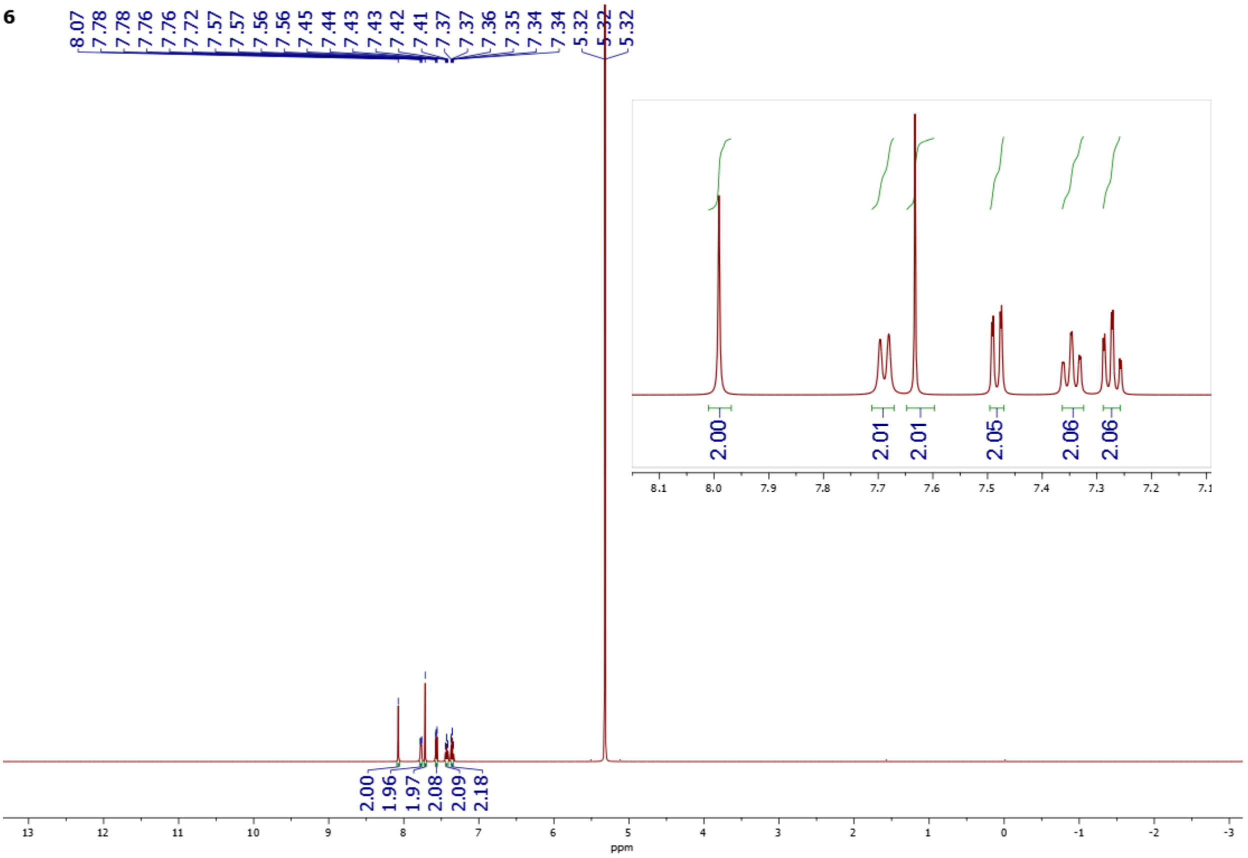

^{13}C NMR (126 MHz, CD_2Cl_2) of [1,1':3',1'':3'',1'''-quaterphenyl]-2,2''',4',4'',6',6''-tris(dithiin) (**6**)

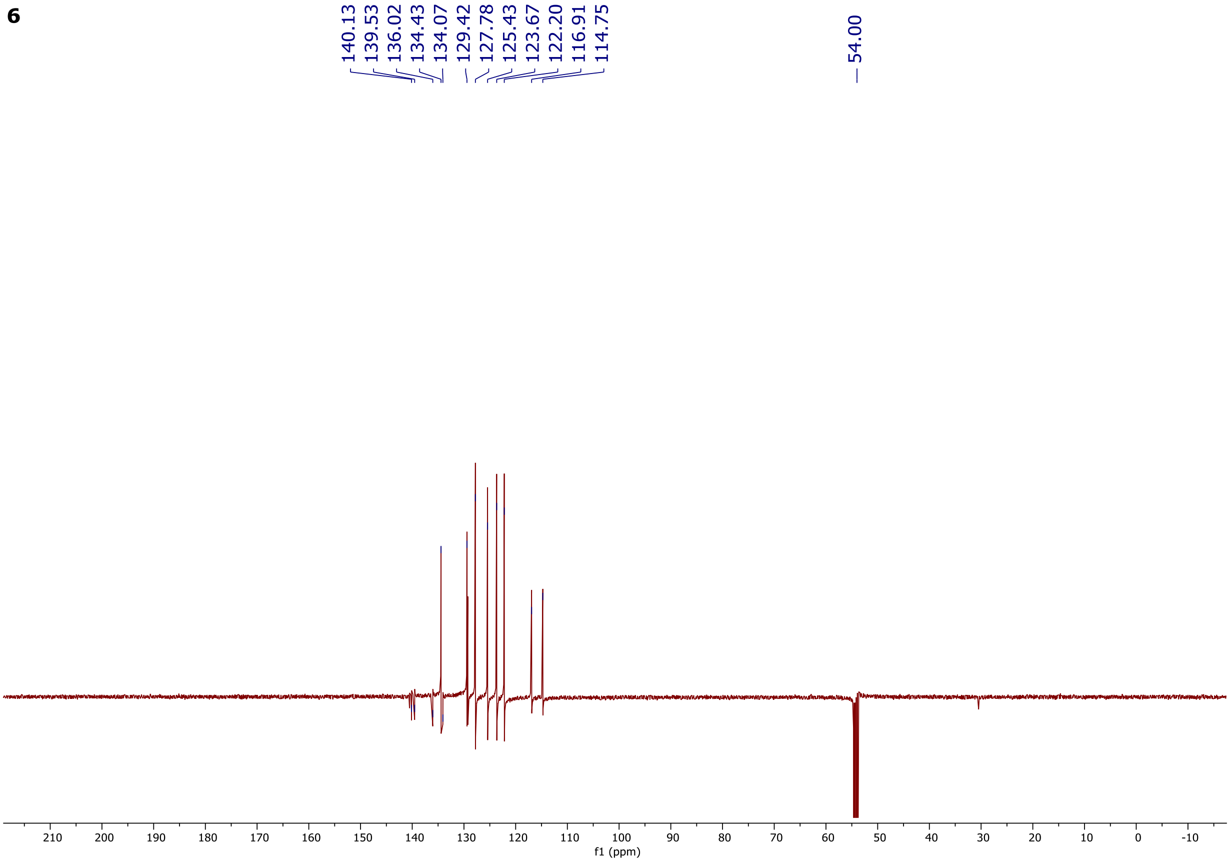

^{1}H NMR (700 MHz, $C_2D_2Cl_4$, 350 K) of [1,1':4',1'':4'',1'''-quaterphenyl]-2,2',2'',2''',5',5''-tris(dithiin) (**7**)

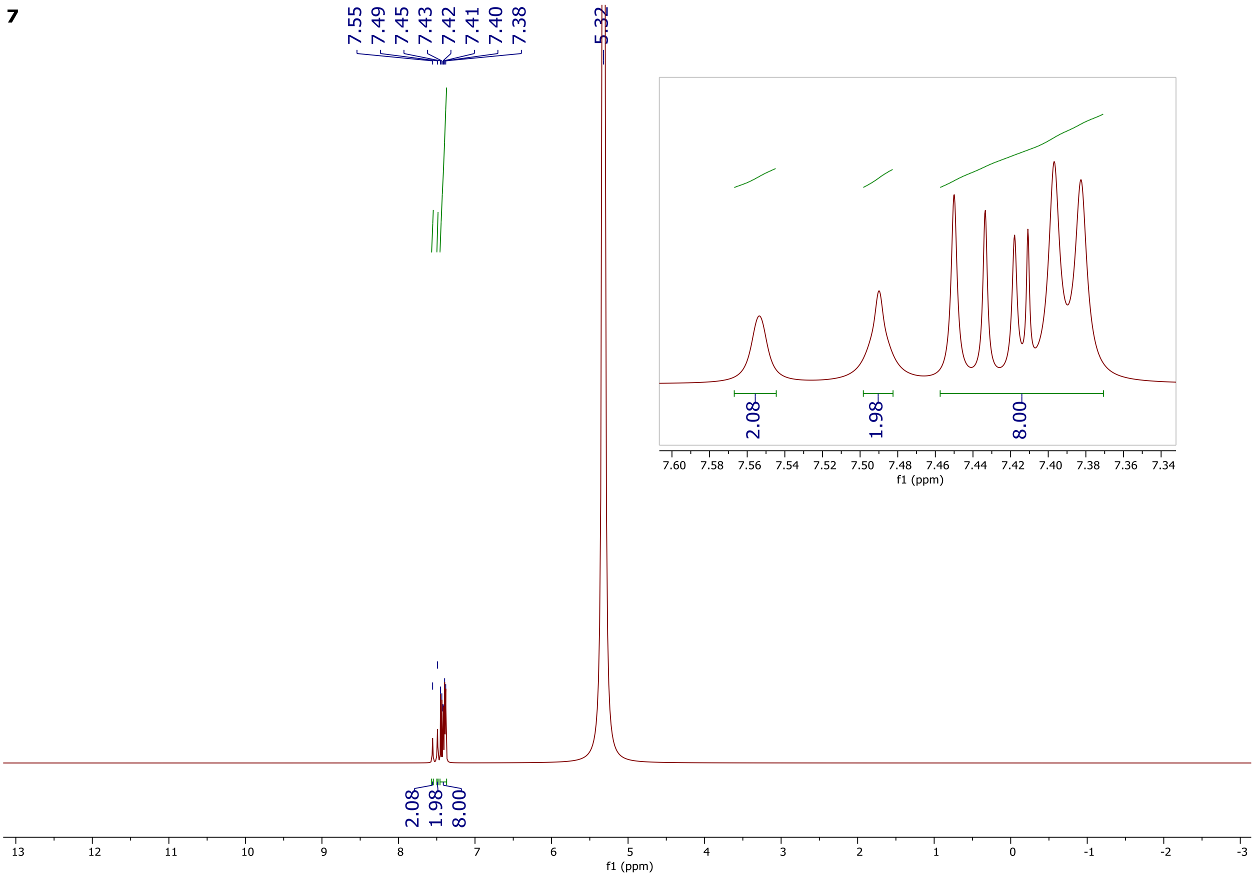

MALDI-ToF spectra of [1,1':4',1'':4'',1'''-quaterphenyl]-2,2',2'',2''',5',5''-tris(dithiin) (**7**)

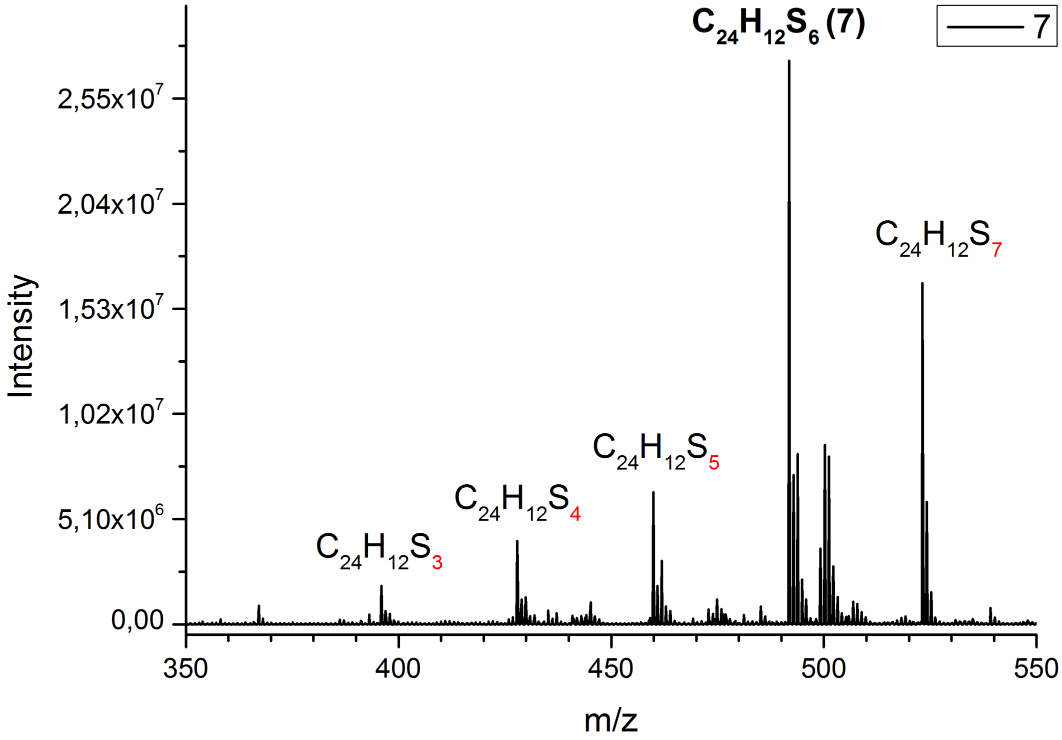

Fragmentation could not be suppressed by adjusting the laser attenuation.

^{1}H NMR (500 MHz, CD_2Cl_2) of 2,2'',4',6'-tetrakis(methylthio)-1,1':3',1''-terphenyl (**9**)

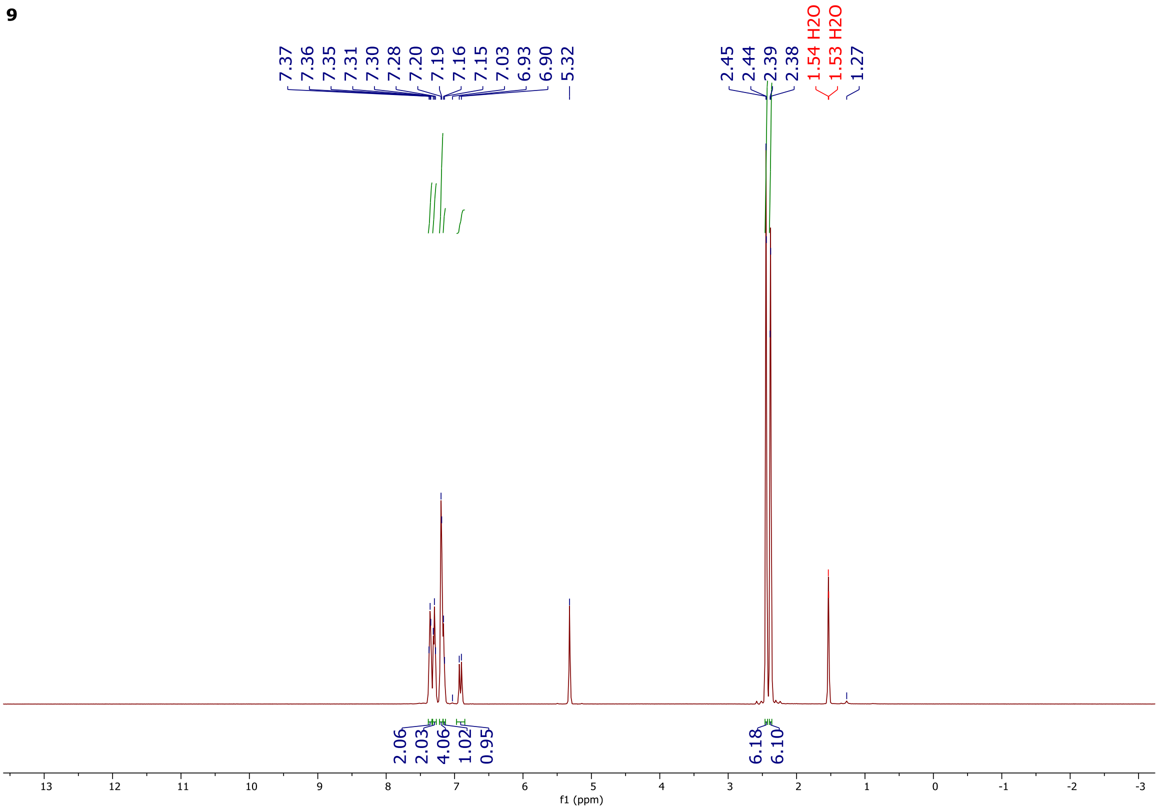

^{13}C NMR (126 MHz, CD_2Cl_2) of 2,2'',4',6'-tetrakis(methylthio)-1,1':3',1''-terphenyl (**9**)

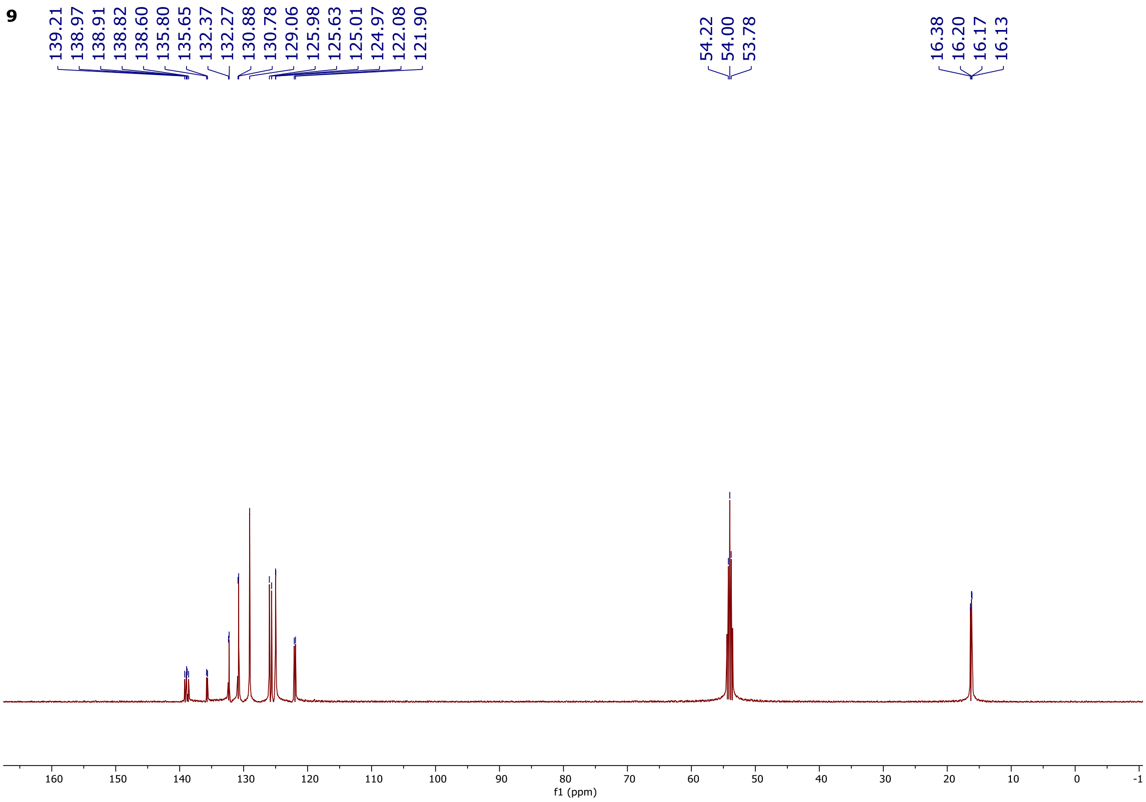

^{1}H NMR (700 MHz, $C_2D_2Cl_4$) of 2,2',2'',5'-T=tetrakis(methylthio)-[1,1':4',1''-terphenyl] (**10**)

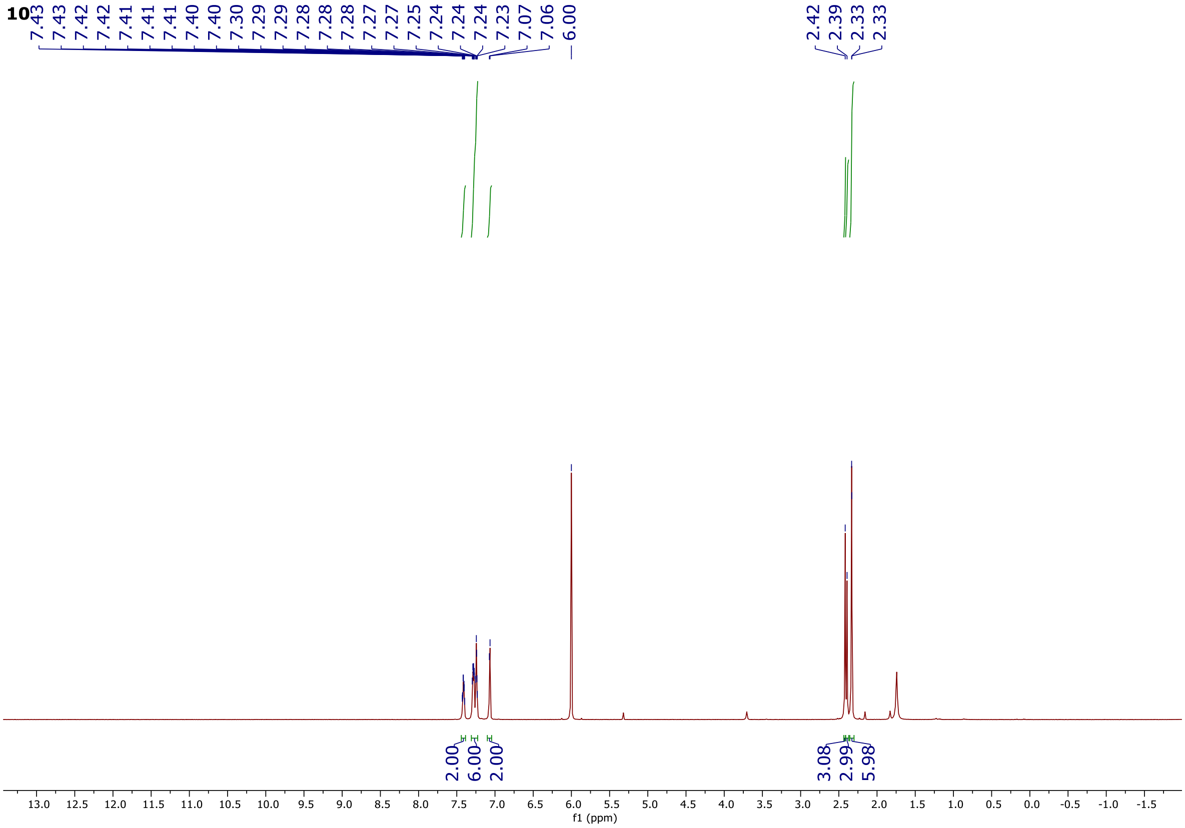

^{13}C NMR (176 MHz, $C_2D_2Cl_4$) of 2,2',2'',5'-tetrakis(methylthio)-[1,1':4',1''-terphenyl] (**10**)

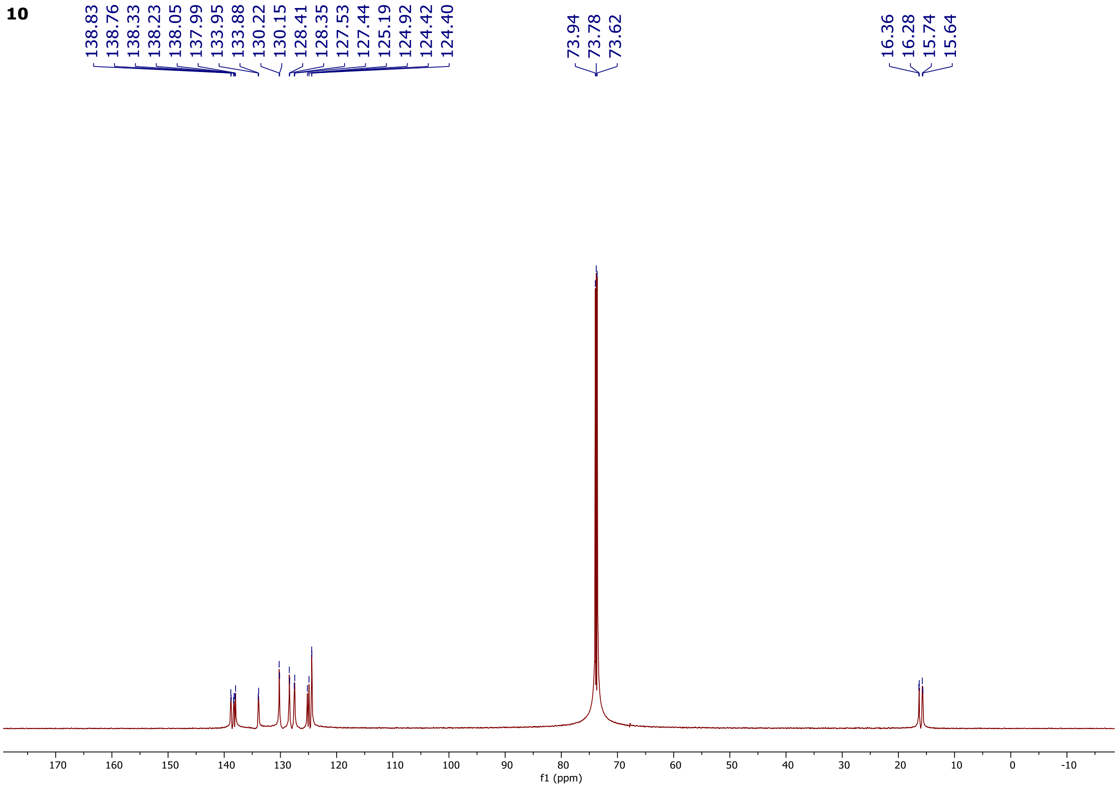

^{1}H NMR (300 MHz, CD_2Cl_2) of 2,2''',4',4'',6',6''-hexakis(methylthio)-[1,1':3',1'':3'',1'''-quaterphenyl] (**11**)

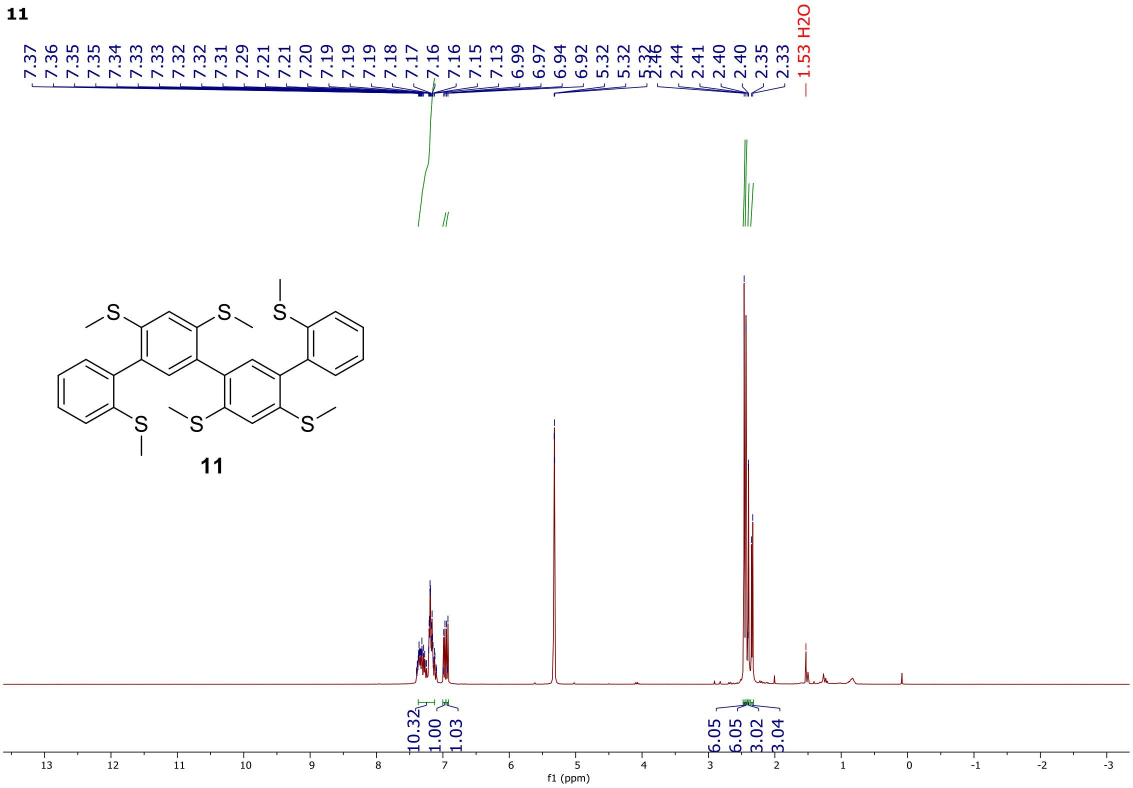

11

^{13}C NMR (126 MHz, CD_2Cl_2) of 2,2''',4',4'',6',6''-hexakis(methylthio)-[1,1':3',1'':3'',1'''-quaterphenyl] (**11**)

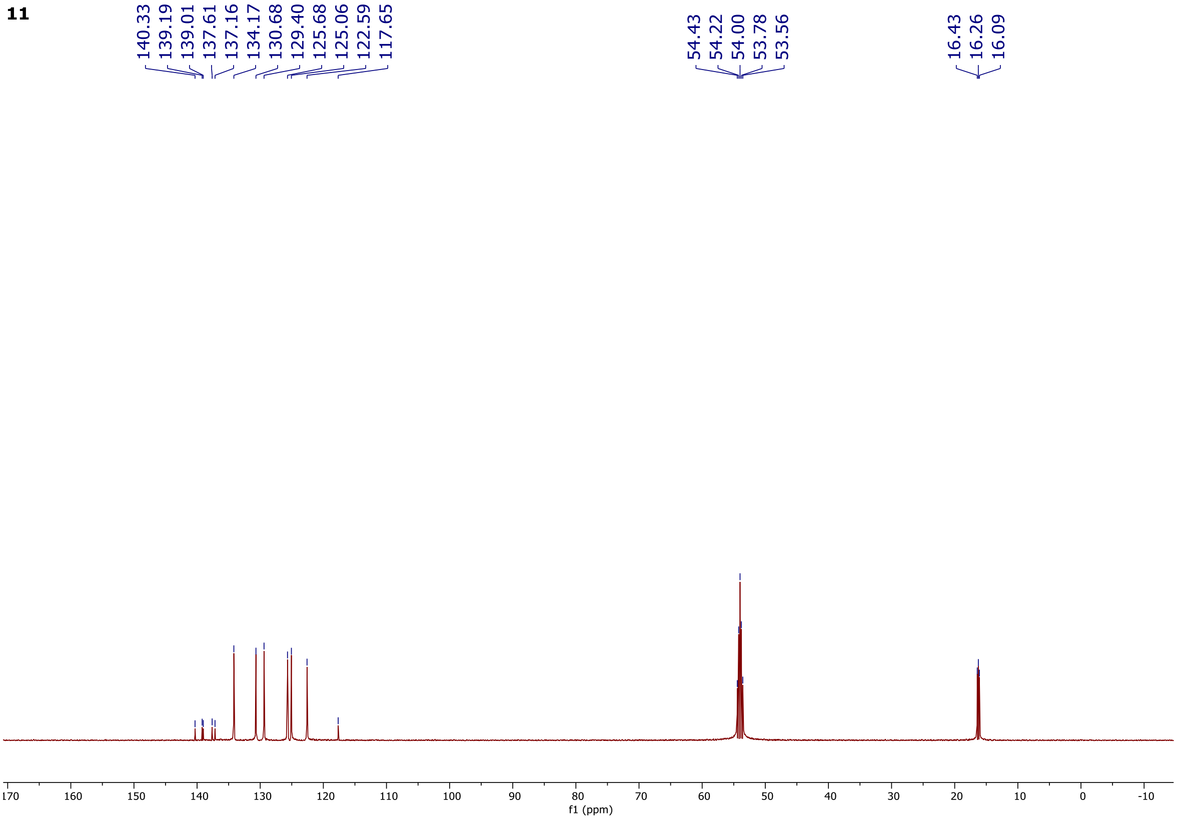

^{1}H NMR (700 MHz, CD_2Cl_2) of 2,2',2'',2''',5',5''-hexakis(methylthio)-[1,1':4',1'':4'',1'''-quaterphenyl] (**12**)

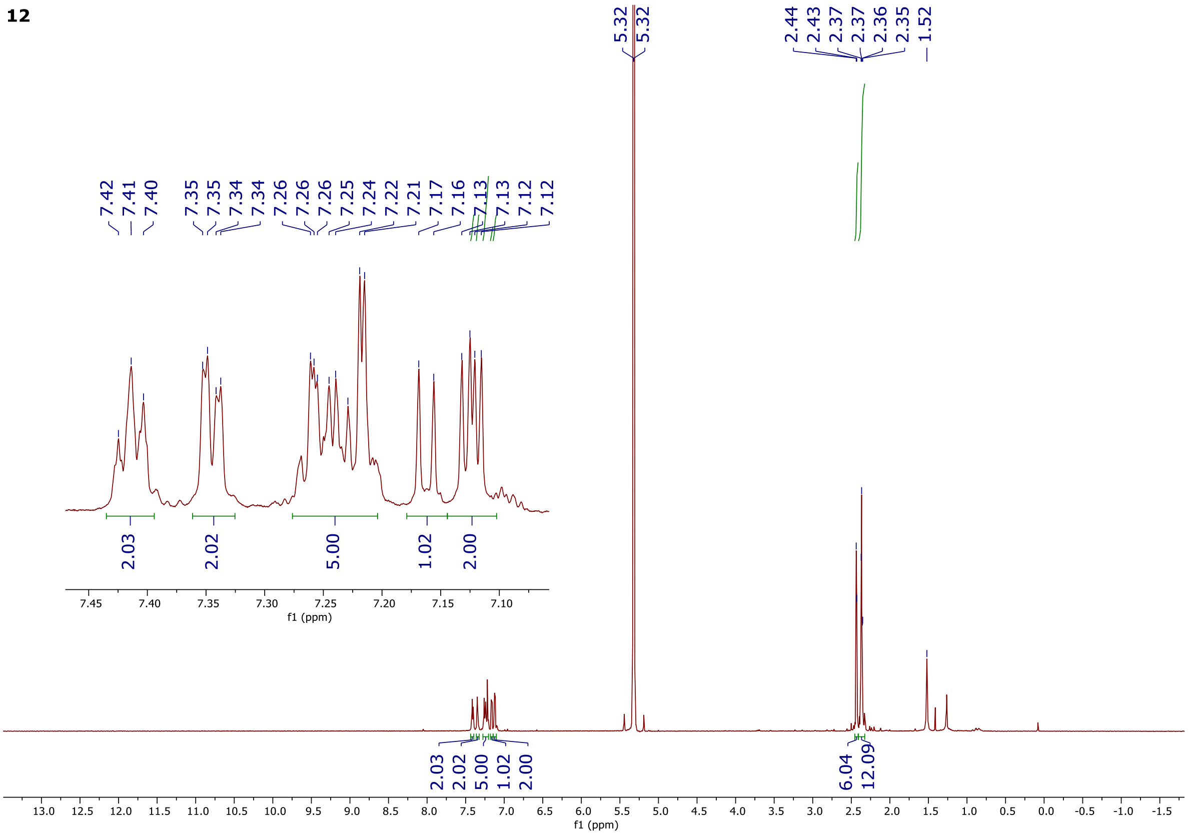

^{13}C NMR (176 MHz, CD_2Cl_2) of 2,2',2'',2''',5',5''-hexakis(methylthio)-[1,1':4',1'':4'',1'''-quaterphenyl] (**12**)

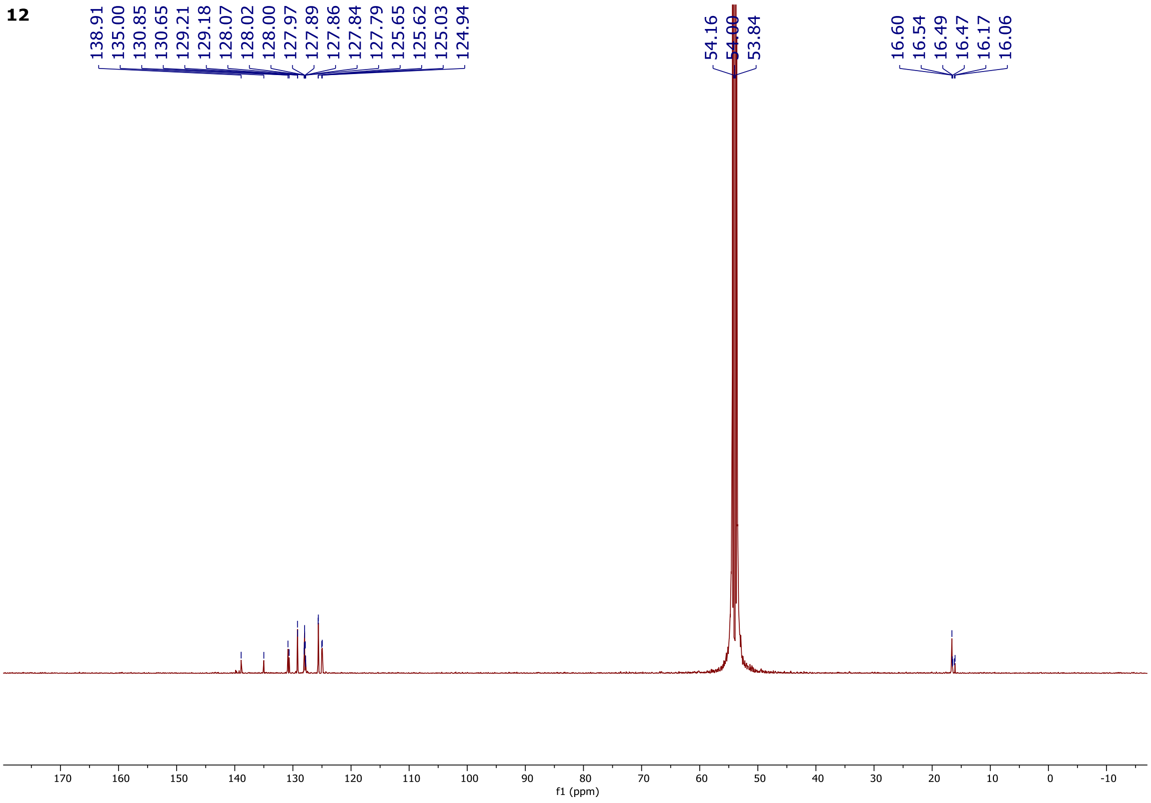

^{1}H NMR (300 MHz, CD_2Cl_2) of 2-{5-bromo-2,4-bis(methylthio)benzene}-4,4,5,5-tetramethyl-1,3,2-dioxaborolane (**17**)

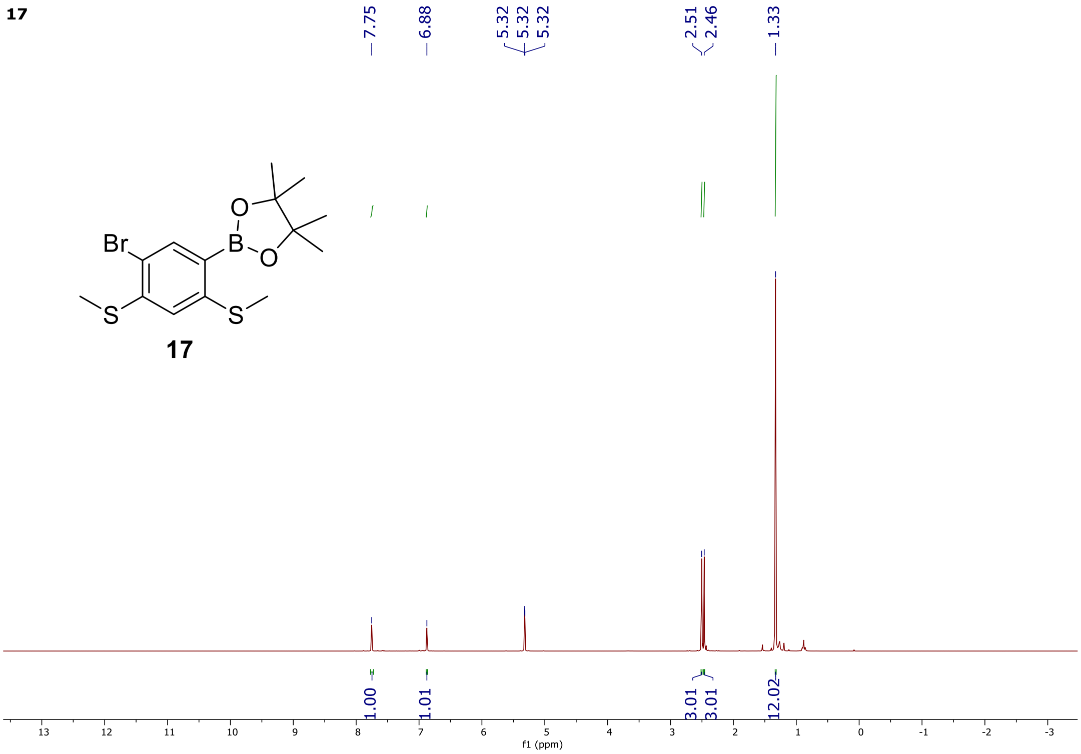

^{13}C NMR (126 MHz, CD_2Cl_2) of 2-{5-bromo-2,4-bis(methylthio)benzene}-4,4,5,5-tetramethyl-1,3,2-dioxaborolane (**17**)

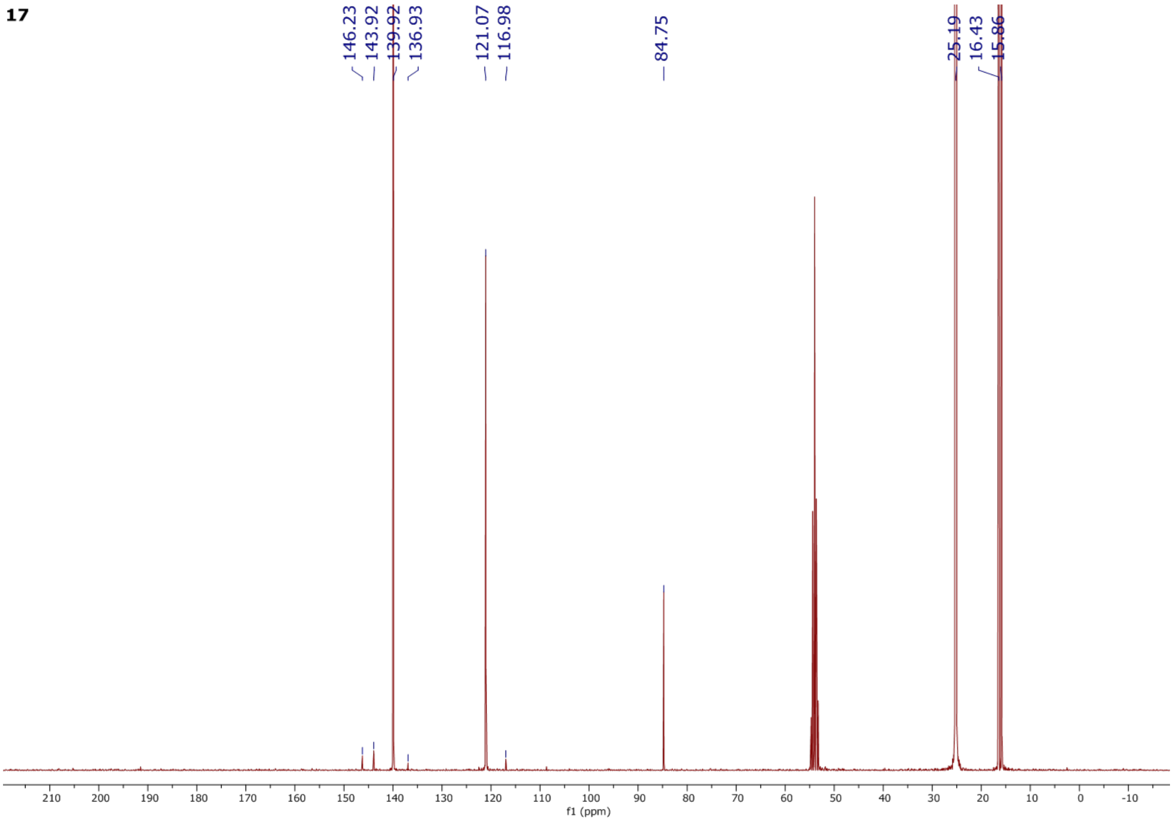

^{1}H NMR (300 MHz, CD_2Cl_2) of 4-bromo-6-iodo-1,3-bis(methylthio)benzene (**18**)

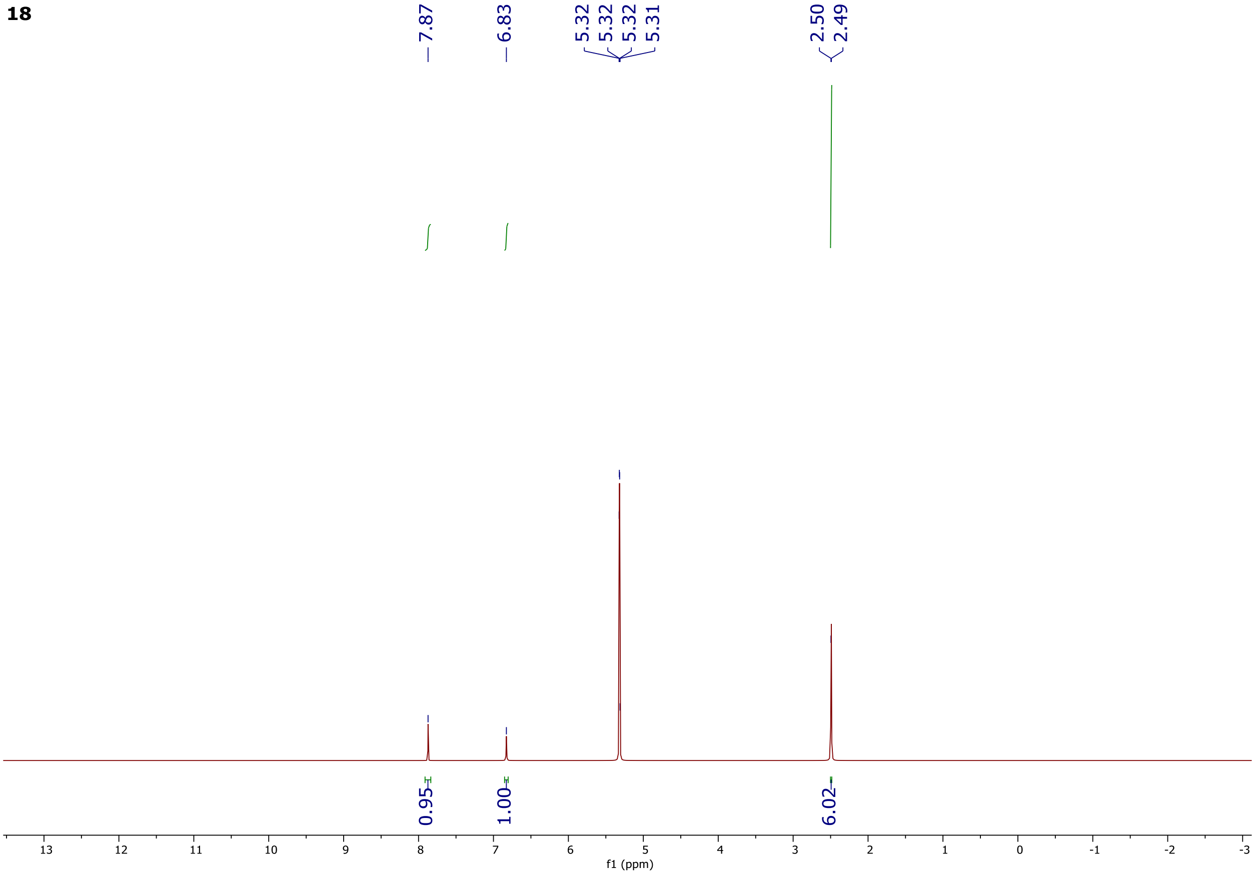

^{13}C NMR (75 MHz, CD_2Cl_2) of 4-bromo-6-iodo-1,3-bis(methylthio)benzene (**18**)

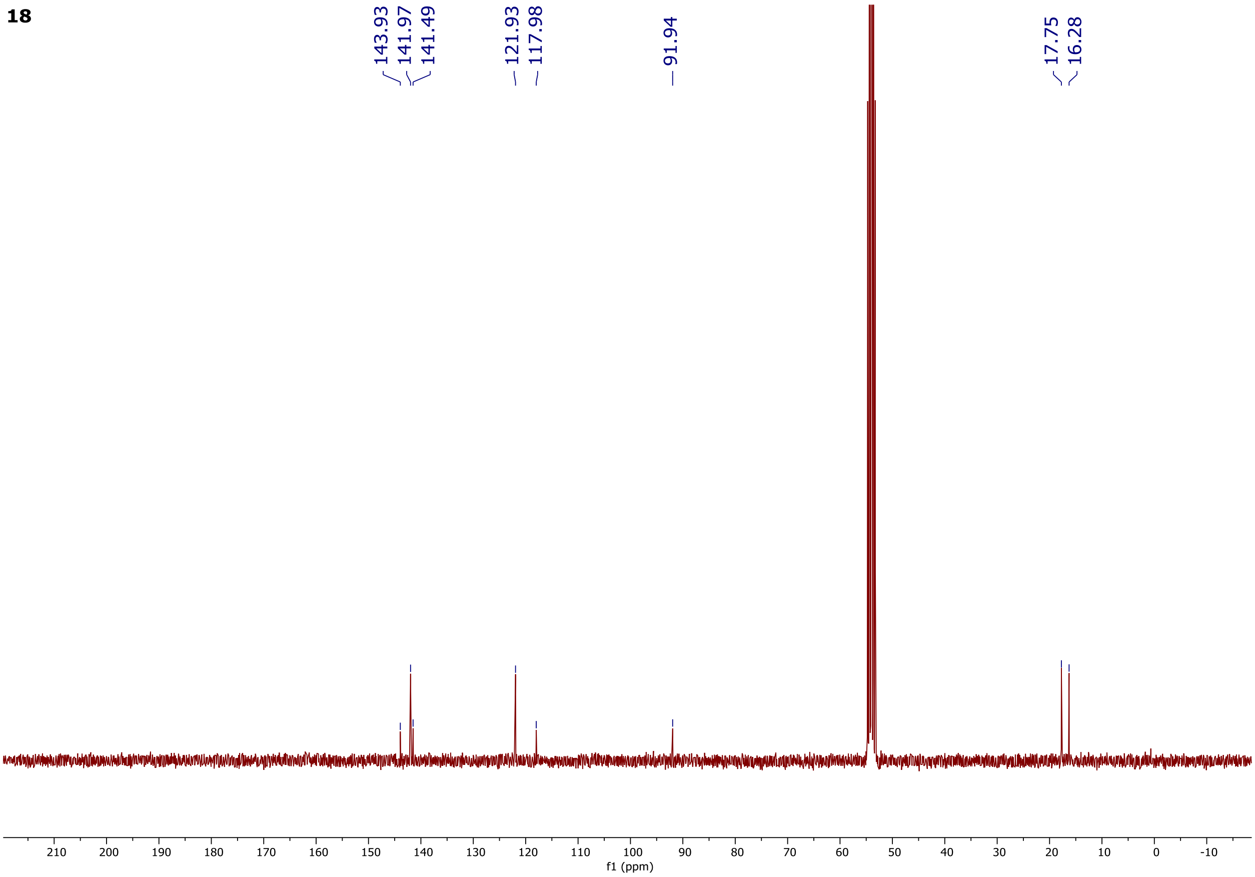

^{1}H NMR (300 MHz, CD_2Cl_2) of 5,5'-dibromo-2,2',4,4'-tetrakis(methylthio)-1,1'-biphenyl (**19**)

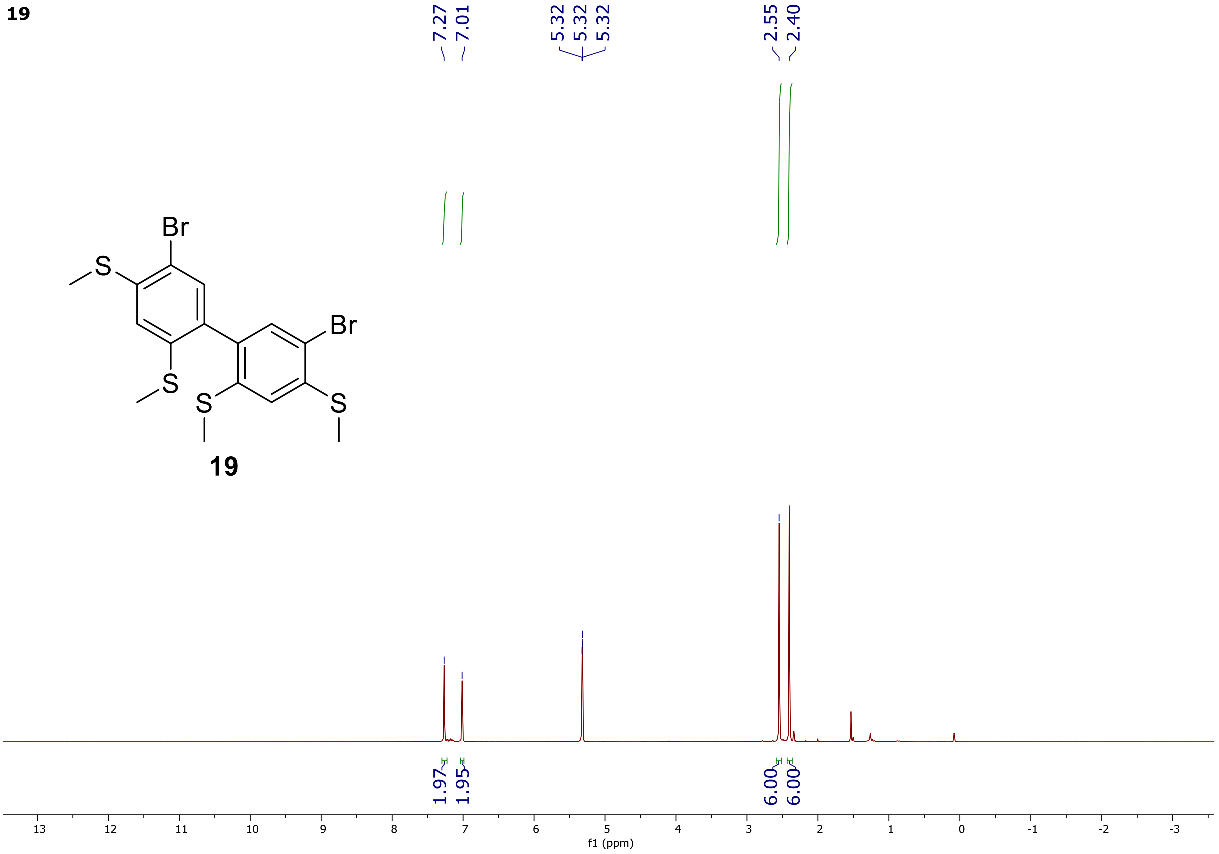

^{13}C NMR (126 MHz, CD_2Cl_2) of 5,5'-dibromo-2,2',4,4'-tetrakis(methylthio)-1,1'-biphenyl (**19**)

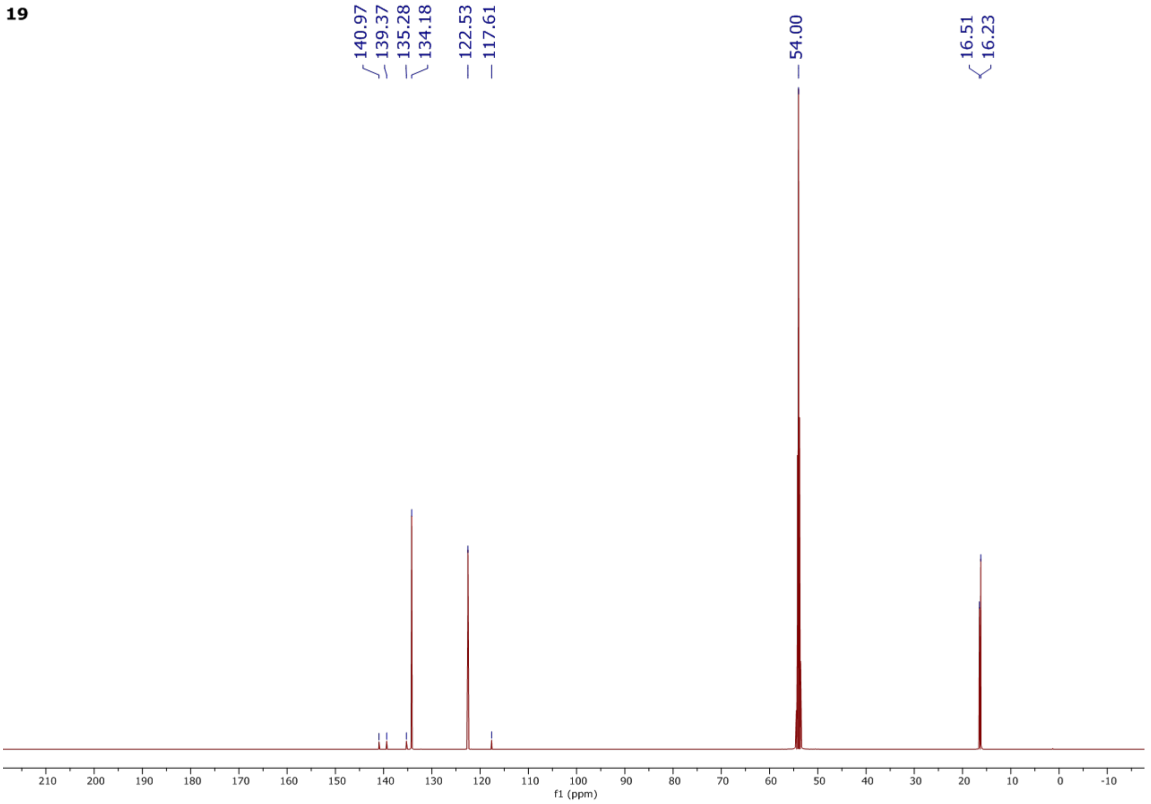

^{1}H NMR (300 MHz, CD_2Cl_2) of 2-bromo-5-iodo-1,4-*bis*(methylthio)benzene (**21**)

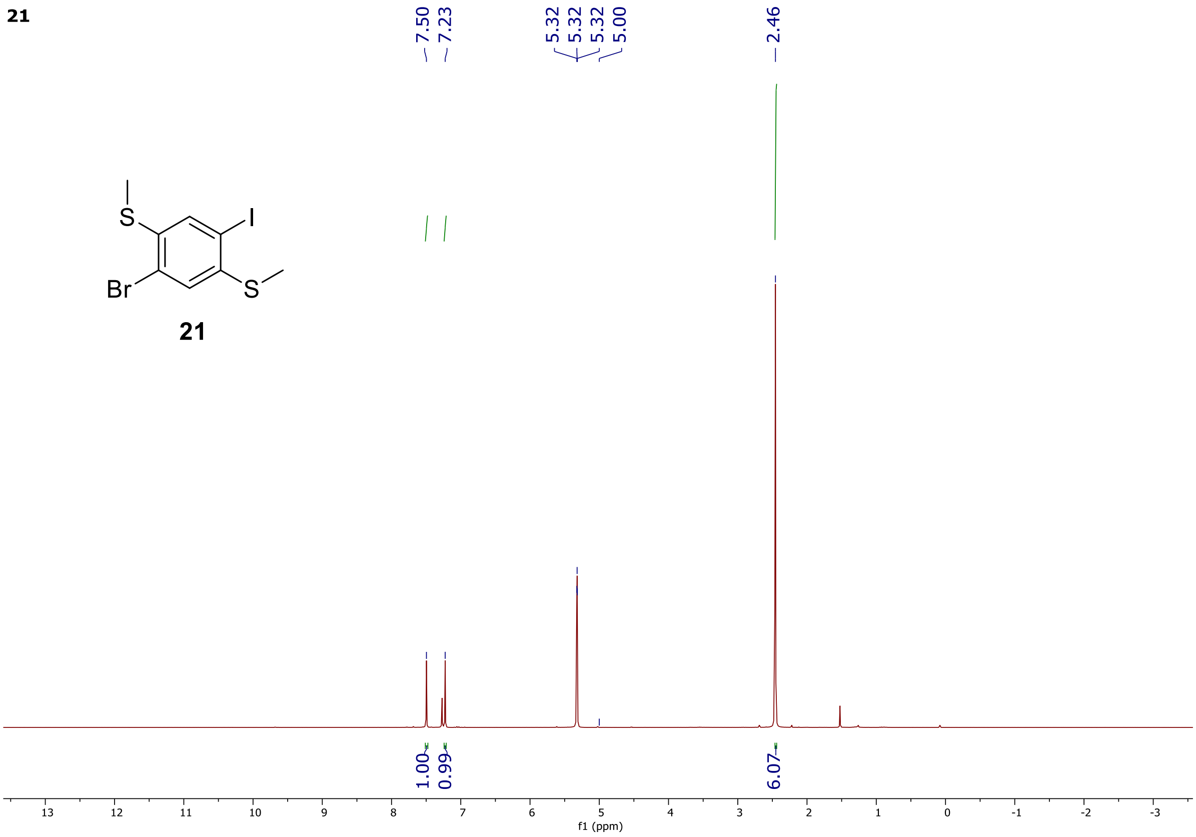

^{13}C NMR (126 MHz, CD_2Cl_2) of 2-bromo-5-iodo-1,4-*bis*(methylthio)benzene (**21**)

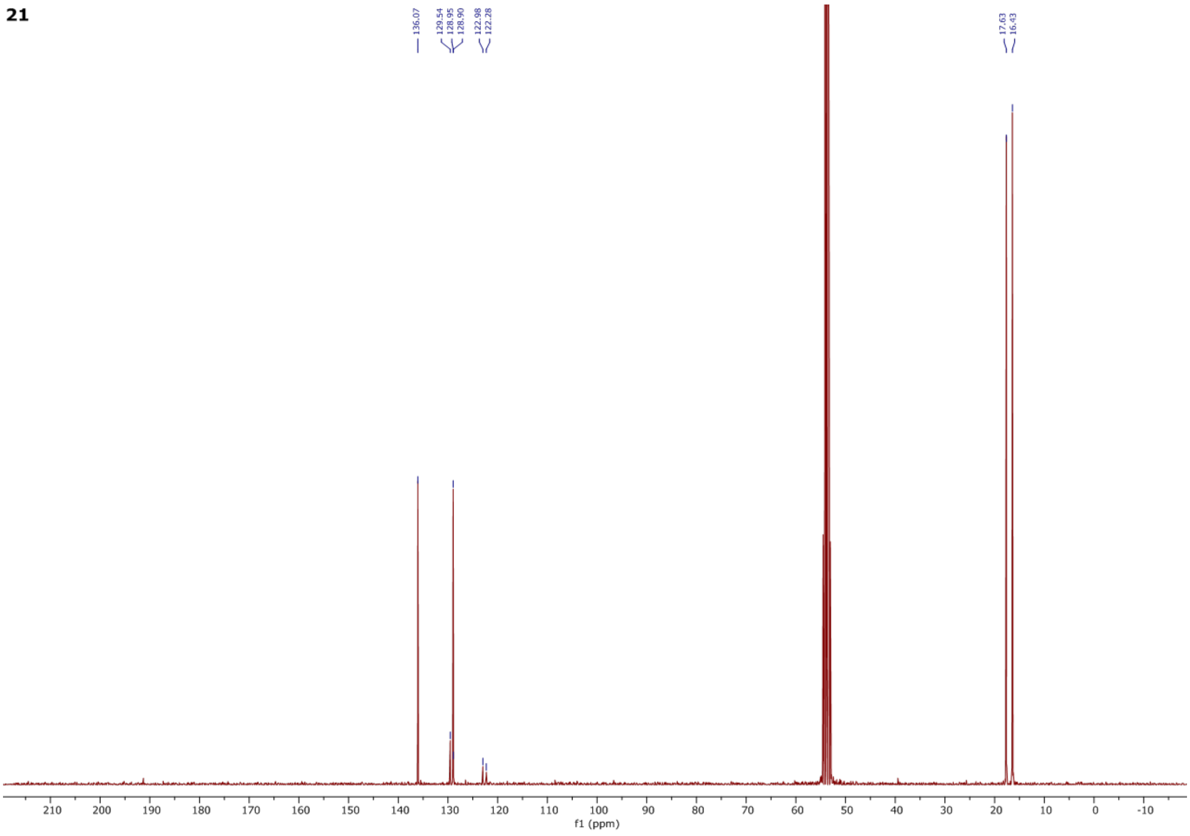

^{1}H NMR (300 MHz, THF-d_8) of 4-bromo-2,5-bis(methylthio)benzene boronic acid (**22**)

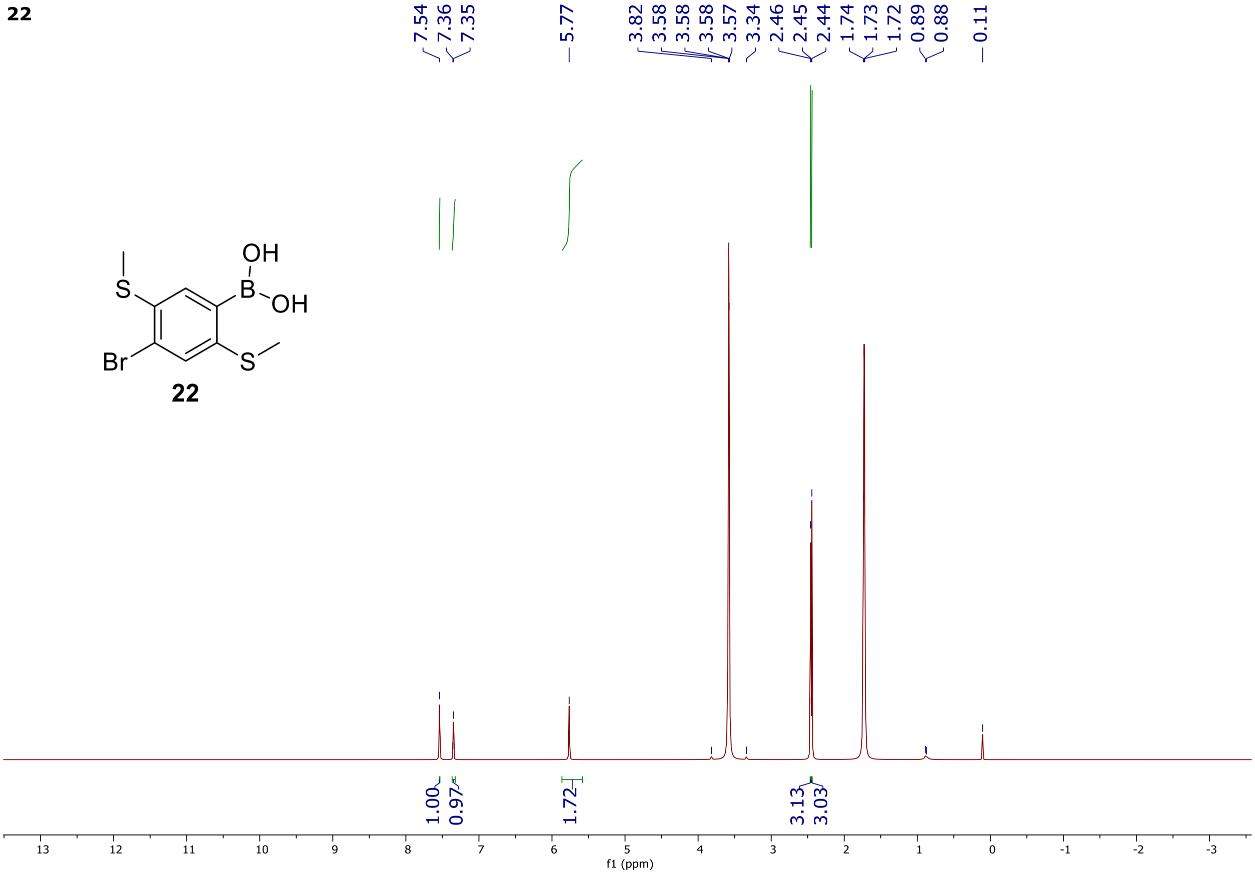

^{13}C NMR (126 MHz, THF-d_8) of 4-bromo-2,5-bis(methylthio)benzene boronic acid (**22**)

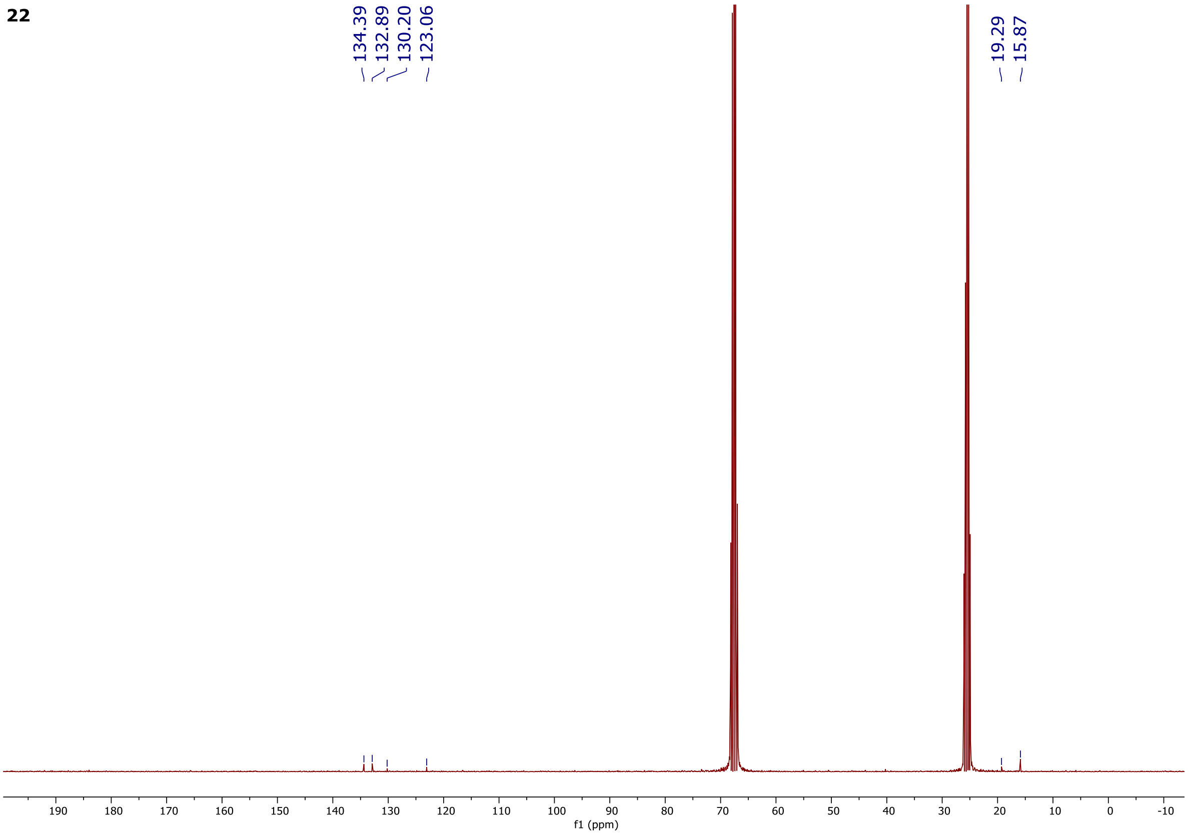

^{1}H NMR (300 MHz, CD_2Cl_2) of 4,4'-dibromo-2,2',5,5'-tetrakis(methylthio)-[1,1'-biphenyl] (**23**)

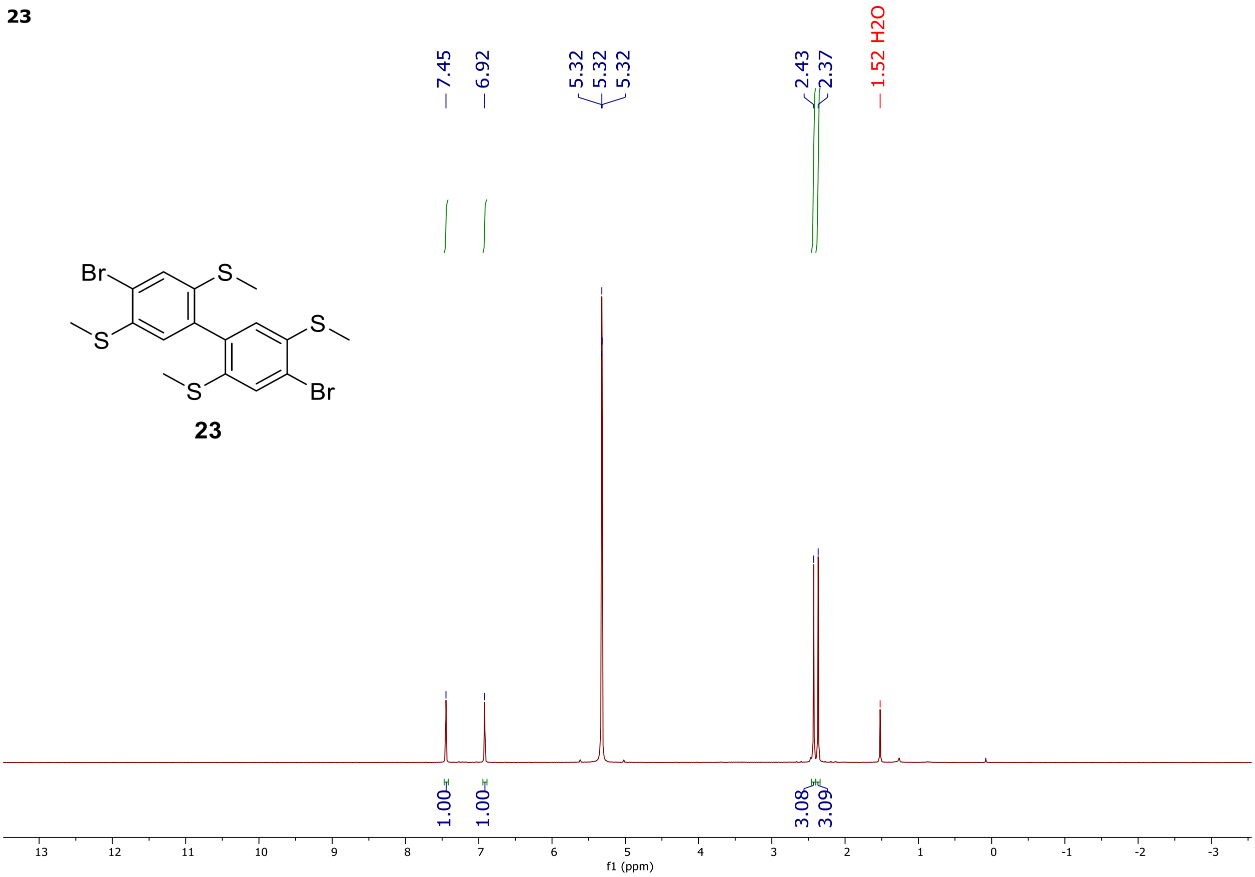

^{13}C NMR (126 MHz, CD_2Cl_2) of 4,4'-dibromo-2,2',5,5'-tetrakis(methylthio)-[1,1'-biphenyl] (**23**)

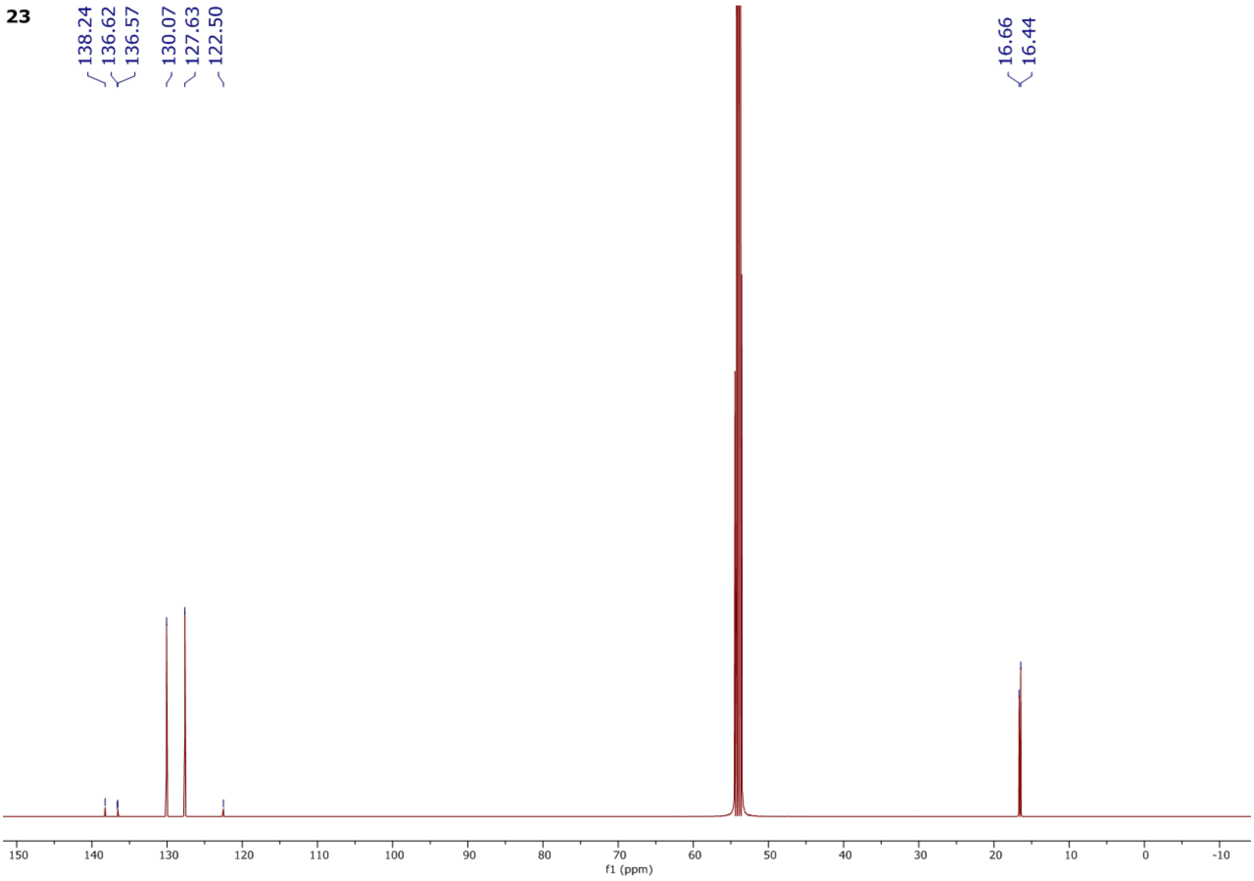

^{1}H NMR (300 MHz, CD_2Cl_2) of dibenzo[*b*,*b*']thieno[3,2-f:4,5-f']-bis[1]benzothiophene (**24**)

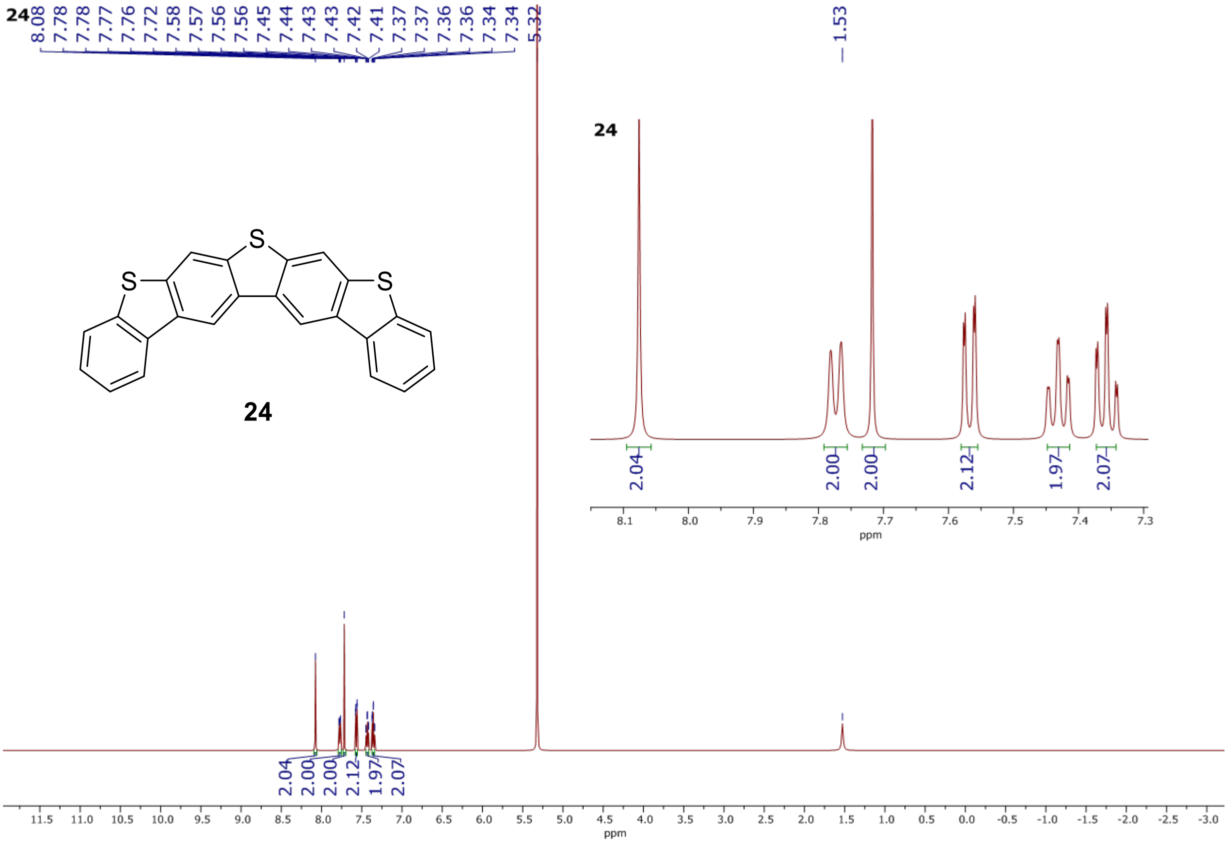

^{13}C NMR (126 MHz, CD_2Cl_2) of dibenzo[*b*,*b*']thieno[3,2-f:4,5-f']-bis[1]benzothiophene (**24**)

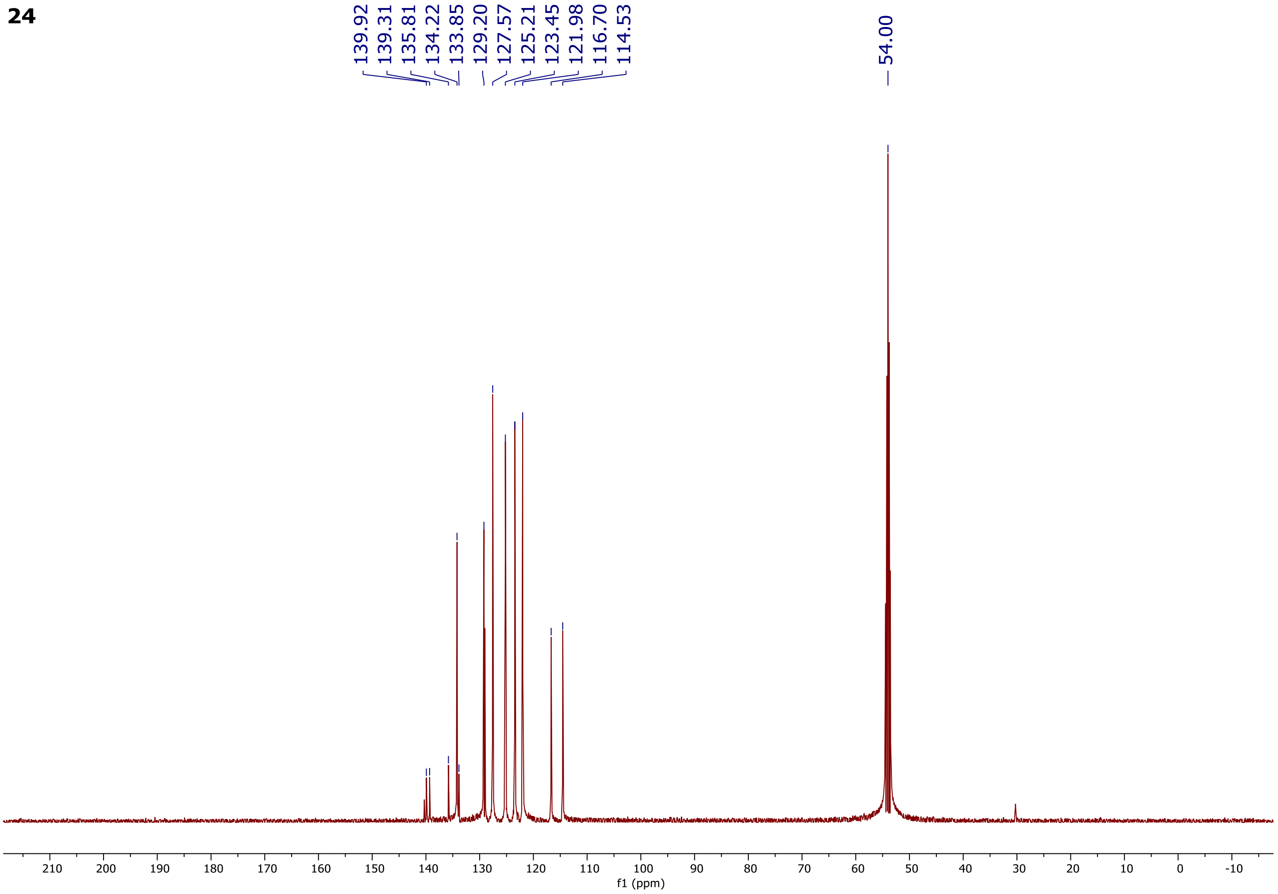

8. References

[1] a) M. Barbero, S. Bazzi, S. Cadamuro, S. Dughera, C. Magistris, P. Venturello, *Synlett* **2010**, 1803-1806; b) J. Buter, D. Heijnen, C. Vila, V. Hornillos, E. Otten, M. Giannerini, A. J. Minnaard, B. L. Feringa, *Angew. Chem. Int. Ed.* **2016**, *55*, 3620-3624.

[2] R. Ferguson, P. S. Nejman, A. M. Z. Slawin, J. D. Woollins, *J. Mol. Struct.* **2017**, *1143*, 405-414.

[3] K. Miyatake, A. S. Hay, F. Mitsuhashi, E. Tsuchida, *Macromolecules* **2001**, *34*, 2385-2388.

[4] P. Gao, X. Feng, X. Yang, V. Enkelmann, M. Baumgarten, K. Müllen, *J. Org. Chem.* **2008**, *73*, 9207-9213.

[5] a) Y. Li, H. Zhan, L. Kong, C. Zhan, Y. Zhou, *Electrochem. Commun.* **2007**, *9*, 1217-1221; b) F. Wang, X. Wu, C. Li, Y. Zhu, L. Fu, Y. Wu, X. Liu, *Energy Environ. Sci.* **2016**, *9*, 3570-3611.
